# Orchestrated control of filaggrin–actin scaffolds underpins cornification

**DOI:** 10.1038/s41419-018-0407-2

**Published:** 2018-03-15

**Authors:** Danuta Gutowska-Owsiak, Jorge Bernardino de La Serna, Marco Fritzsche, Aishath Naeem, Ewa I. Podobas, Michael Leeming, Huw Colin-York, Ryan O’Shaughnessy, Christian Eggeling, Graham S. Ogg

**Affiliations:** 1 MRC Human Immunology Unit, Weatherall Institute of Molecular Medicine, Radcliffe Department of Medicine, University of Oxford, Oxford, OX3 9DS UK; 2 Institute of Biotechnology UG, Intercollegiate Faculty of Biotechnology of University of Gdańsk and Medical University of Gdańsk, 80-307 Gdańsk, Poland; 3 Research Complex at Harwell, Central Laser Facility, Rutherford Appleton Laboratory Science and Technology Facilities Council, Harwell-Oxford, Didcot, OX11 0FA UK; 4 Kennedy Institute of Rheumatology, Nuffield Department of Orthopaedics, Rheumatology and Musculoskeletal Sciences, Oxford, OX3 7FY UK; 5 Immunobiology, UCL Great Ormond Street Institute of Child Health, London, WC1N 1EH UK; 6 Institute of Biochemistry and Biophysics, Polish Academy of Sciences, Pawińskiego 5a, 02-106 Warsaw, Poland; 7 Centre for Cell Biology and Cutaneous Research, Blizard Institute, Barts and the London School of Medicine and Dentistry, Queen Mary University of London, London, E1 2AT UK; 8 Institute of Applied Optics, Friedrich-Schiller-University Jena, Max-Wien Platz 4, 07743 Jena, Germany; 9 Leibniz Institute of Photonic Technology e.V., Albert-Einstein-Straße 9, 07745 Jena, Germany

## Abstract

Epidermal stratification critically depends on keratinocyte differentiation and programmed death by cornification, leading to formation of a protective skin barrier. Cornification is dynamically controlled by the protein filaggrin, rapidly released from keratohyalin granules (KHGs). However, the mechanisms of cornification largely remain elusive, partly due to limitations of the observation techniques employed to study filaggrin organization in keratinocytes. Moreover, while the abundance of keratins within KHGs has been well described, it is not clear whether actin also contributes to their formation or fate. We employed advanced (super-resolution) microscopy to examine filaggrin organization and dynamics in skin and human keratinocytes during differentiation. We found that filaggrin organization depends on the cytoplasmic actin cytoskeleton, including the role for α- and β-actin scaffolds. Filaggrin-containing KHGs displayed high mobility and migrated toward the nucleus during differentiation. Pharmacological disruption targeting actin networks resulted in granule disintegration and accelerated cornification. We identified the role of AKT serine/threonine kinase 1 (AKT1), which controls binding preference and function of heat shock protein B1 (HspB1), facilitating the switch from actin stabilization to filaggrin processing. Our results suggest an extended model of cornification in which filaggrin utilizes actins to effectively control keratinocyte differentiation and death, promoting epidermal stratification and formation of a fully functional skin barrier.

## Introduction

Orchestrated keratinocyte differentiation and death are indispensable for the formation of the *stratum corneum*, the outermost layer of the epidermis which confers barrier function to both physical insult and infection. During differentiation, keratinocytes withdraw from the cell cycle and move suprabasally throughout the layers up to the stratum granulosum of the epidermis^1^, where they undergo a modified form of programmed cell death (cornification)^2^. Cornification involves sequential expression of epidermis-specific proteins such as keratins, loricrin, involucrin and filaggrin, changes in the stiffness of cellular membranes, aggregation of intermediate filaments, release of lamellar bodies, removal of organelles, and formation of an insoluble “cornified envelope” (reviewed in ref.^3^). Aberration of cornification results in clinical consequences, ranging from the propensity toward infections, formation of a mechanically fragile barrier, and skin dehydration to allergen sensitization and inflammation^4–8^.

Filaggrin (filament-aggregating protein) is a key component in epidermal cornification and barrier formation^9^. Multiple filaggrin units are posttranslationally hydrolyzed from a large precursor protein (profilaggrin) during keratinocyte differentiation^10^. Profilaggrin is observed in a form of dense protein aggregates, “keratohyalin granules” (KHGs)^11,12^; the main source of biologically active filaggrin monomers. Filaggrin release from KHGs into the cytoplasm leads to binding and collapse of intermediate filaments initiating rapid cell death^13^; consequently, tight control of the monomer release is critical to support skin barrier function. Consistently, null mutations in the *filaggrin* gene (*FLG)* strongly associate with moderate to severe atopic dermatitis (AD)^14,15^. Dysregulation of expression or profilaggrin processing leads to reduced barrier integrity, observed in skin diseases, animal models, and in vitro^5,16–22^. Moreover, the presence of *FLG* mutation also predisposes to additional allergic manifestations (asthma, rhinitis, food, and contact allergy)^15,23–26^.

Intracellular filaggrin is known to be involved in the stimulation of keratinocyte differentiation via N-terminal domain signaling^27,28^, having effects on the cortical actin and associating keratin filaments, causing their aggregation into bundles^13,29–31^. However, despite the well-established role of filaggrin in skin physiology and disease, little is known about the organization of the protein within the cell, mechanisms governing its release from the granules, and interactions with the cytoplasmic skeleton during keratinocyte differentiation and progression of cornification. These aspects may provide answers with potential for clinical applications and require clarification.

One factor involved in keratinocyte differentiation and progression of cornification might be the actin cytoskeleton. Cortical actin networks are dynamic structures comprising filaments undergoing continuous turnover and growth at barbed ends and shrinkage at pointed ends^32^. The filaments are cross-linked and redistributed by the action of molecular motors such as myosin-II^33^. Two filament subpopulations compose the cortex in eukaryotic cells^34,35^: long formin-nucleated actin filaments and short actin filaments nucleated by the Arp2/3 complex. Arp2/3-nucleated F-actin accounts for the majority of the total F-actin in various cell types; the small (10–20% of the total) fraction of formin-mediated F-actin predominantly participates in force generation during transport of molecular components and adjustment of mechanical properties of the cells.

However, additional proteins can also be involved. Specifically, the heat shock protein 27 (HspB1), a molecular chaperone implicated in cellular stress resistance, may play an important role. HspB1 is known to bind and stabilize actin^36–38^; on the other hand, HspB1 is also present in KHGs of terminally differentiated keratinocytes, where it is believed to facilitate filaggrin processing. HspB1 interaction with filaggrin has been shown to specifically depend on the activity of AKT serine/threonine kinase 1 (AKT1), a cellular signaling mediator expressed late during epidermal terminal differentiation^39^. The mechanism involving actin, HspB1, and AKT1 could therefore provide an important link between KHGs and the cytoskeleton, potentially critical during cornification. However, it mostly remained elusive until now because of a profound lack of imaging technology allowing to monitor cytoskeleton dynamics and filaggrin.

Here, we overcome this limitation by employing advanced imaging, including super-resolution STED microscopy, and identified multiple events leading to cornification of human keratinocytes. We collectively describe these as “granule maturation”; they comprise morphological changes in the shape, as alignment, aggregation, and nucleus-directed migration of the KHGs. We reveal the involvement of muscle α- and non-muscle β-actin, forming a core and scaffold-like structures associated with the granules, respectively. Functional experiments with actin-specific inhibitors highlighted the role of these structures in supporting granule shape and integrity, and regulating important aspects of the cornification process, i.e., membrane stiffness. We show an AKT1-mediated switch of the binding preference of HspB1 from actin to filaggrin, likely facilitating the dissolution of the actin cytoskeleton and subsequent filaggrin processing. Our results point to an extended model of keratinocyte differentiation in which the “profilaggrin/filaggrin system”, already known to include accessory proteins (e.g., processing enzymes), additionally employs actin microfilaments to effectively control KHG maturation and integrity, thus preventing premature progression of cornification and cell death.

## Results

### Maturation of filaggrin-containing KHGs during keratinocyte differentiation

**Fig. 1:**
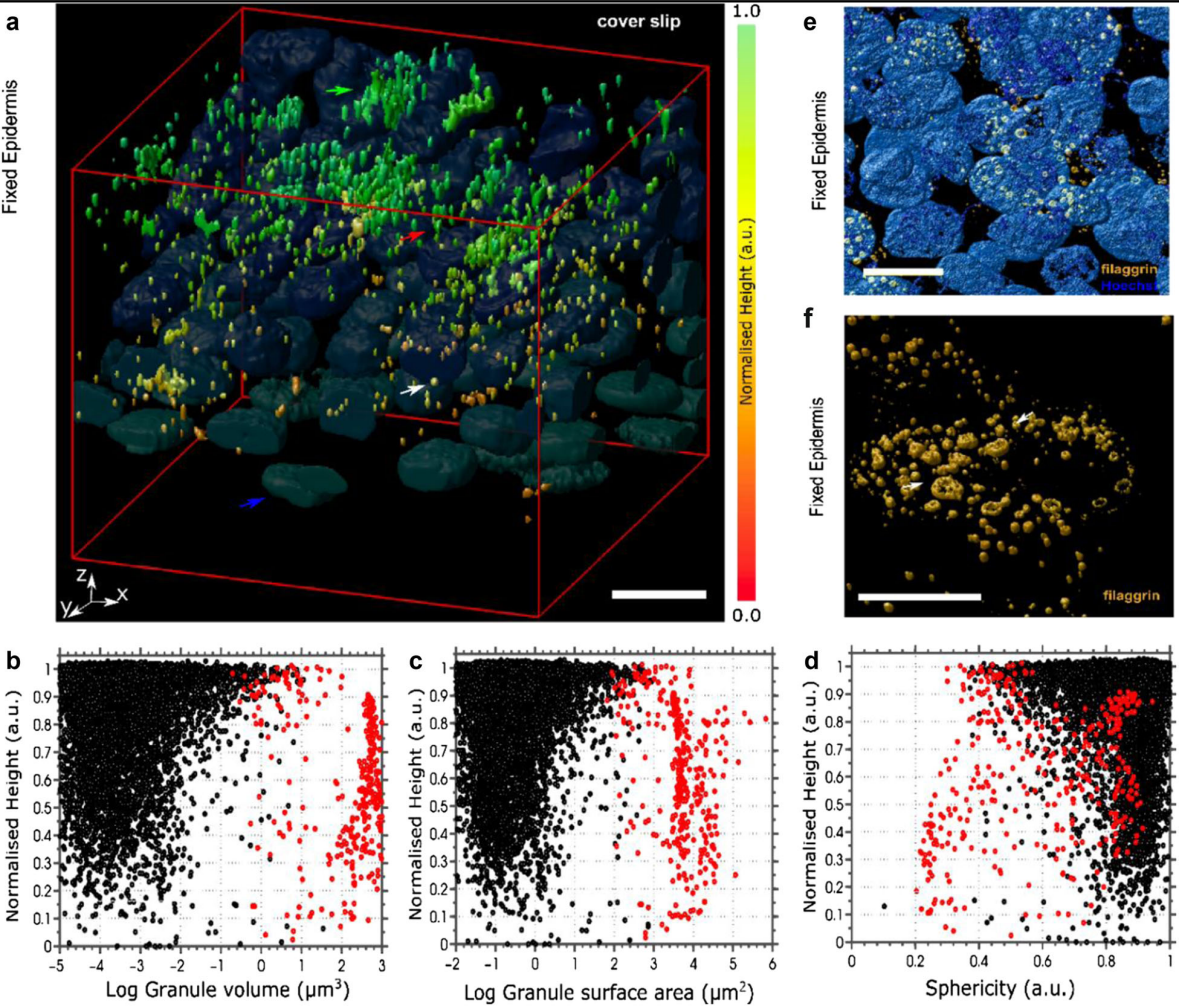
Filaggrin-containing keratohyalin granules undergo shape alterations and aggregation during formation of stratified epidermis. **a** 3D scanning confocal images of fixed epidermal sheets from healthy donors harvested by dispase isolation and stained with anti-filaggrin antibody (G-20 primary and Alexa Fluor 488 secondary antibody). The color scale from red to orange depicts the normalized height relative to the bottom of the epidermis (the most upper epidermal layers at the microscope cover glass in green). Nuclei (blue and representative blue arrow) visualized by Hoechst. The red arrow indicates a representative elongated granule in the mid-layer. The green arrow indicates a representative granule aggregate in the uppermost layer. Scale bar 10 µm; microscope cover glass at the top. The data are representative of eight donors. **b**–**d** Geometric analysis of granule distribution within the epidermis based on topological parameters: volume (**b**), surface area (**c**), and sphericity (1 marks round granules while values <1 indicate elongated shapes) (**d**) relative to normalized height (relative to the bottom of the epidermis). Black dots indicate granules smaller than 3 µm in axial length and red dots indicate granules larger than 3 µm in length; *n* = 9170 granules in 120 cells in *N* = 8 donors. **e**, **f** Magnifications of 3D scanning confocal images of epidermal sheets (as in **a**) highlighting the morphology of ring-shaped keratohyalin granules (orange; arrows): **e** scale bar 10 µm; **f** scale bar 5 µm and nuclei stain removed for clarity. The figure is representative of five donors

Since differentiation processes during stratification and *stratum corneum* formation are complex and involve multiple organellar modifications^2^, we hypothesized that changes in filaggrin-containing KHGs are also inevitable. To test this, we first immunolabeled filaggrin in epidermal sheets from healthy donors (*N* = 8; ex vivo condition). In order to visualize the stages of KHGs, from nascent to terminal and to ensure we were not missing any of the filaggrin^+^ signal due to inaccessibility of monoclonal antibody epitopes (e.g., due to processing or 3D fold of profilaggrin within the granules), we chose a polyclonal anti-filaggrin antibody (G-20). We have validated the reagent by colocalization studies with other anti-filaggrin antibodies (monoclonal N-terminal-specific 15C10 and polyclonal N-terminal site-specific H-300) and showed the same staining pattern (Figure S1). We imaged KHGs by confocal microscopy and carried out 3D reconstruction to spatially localize them with respect to their position within the epidermis, and to quantify their geometric properties (volume, surface area, and sphericity) (Fig. 1) and observed increased granule expression toward the uppermost layers of the epidermal tissue (Fig. 1a), in line with differentiation progression. We found a small pool of granule aggregates, detected as structures more than 3 µm in axial length (representing 4.5% of the total granules; pooled data; red dots) and a large pool of smaller single KHGs with lengths <3 µm (black dots; see also Figure S2). While the larger aggregates maintained their geometrical parameters across all epidermal layers, smaller KHGs increased in number and elongated toward the outer layer (Fig. 1b–d and Figure S2). Moreover, some of the KHGs adapted a ring-like morphology in the uppermost layers (Fig. 1e, f). The occurrence of large aggregates in the adjacent epidermal locations (subjected to the same staining conditions), as well as of “open” half-ring KHGs (Fig. 1e, f) excluded the possibility that these structures are artifacts due to incomplete antibody penetration into large structures. Moreover, the ring-like granules presented a striking similarity to the, largely overlooked, ring-like KHGs detected previously by the (antibody-independent) electron microscopy of the skin and oral mucosa^40–42^, further validating our findings.

### KHG single-cell dynamics in keratinocytes

We next switched to a controllable well-established in vitro “calcium-switch” model to investigate changes in KHG morphology within single cells, using cultured NHEKs (normal human epidermal keratinocytes). In these experiments, calcium concentrations corresponded to the physiological gradient present in the epidermis, known to stimulate keratinocyte differentiation^43^. We first employed this model to visualize filaggrin within KHGs at defined calcium levels [Ca^2+^] = (0.06–5.0 mM). Typically, cells positive for filaggrin KHGs are scarce in cultures under proliferative (low calcium) conditions. Still, a small fraction of cells (~1–2%, depending on the donor and passage) express KHGs due to contact-dependent signals from the neighboring cells; these were analyzed further (Figure S4A).

**Fig. 2:**
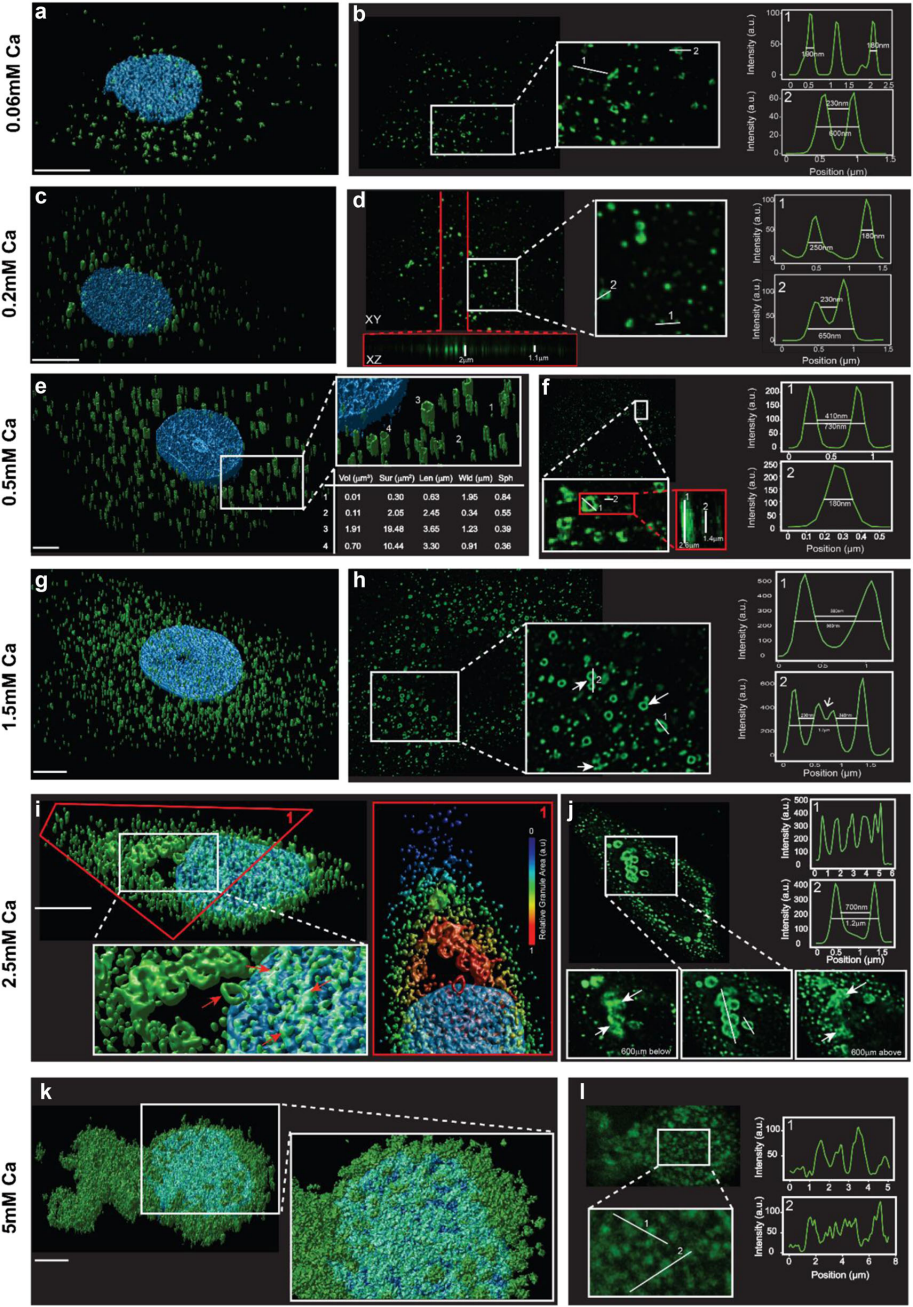
Filaggrin-containing granules undergo shape changes during calcium-induced keratinocyte differentiation in vitro. **a**–**l** 3D (left panels, **a**, **c**, **e**, **g**, **i**, **k**) and 2D (selected *X*–*Y* planes; right panels, **b**,** d**,** f**,** h**, **j**, **l**) scanning confocal images of fixed normal human epidermal keratinocytes (NHEKs) subjected to “calcium switch” at different calcium concentrations [Ca^2+^] = 0.06–0.5 mM (as labeled on the left) to promote their differentiation (granules immunostained with G-20 anti-filaggrin antibody (green) and nuclei with Hoechst (blue)). Scale bar 5 µm. White-bordered insets: magnifications of volumes or areas marked as white boxes in the respective overview images (in **j**, two additional insets are given as the 2D images of the same area at different axial planes as indicated). Red-bordered insets in the right panels: *x*–*z* axial zoom-ins along red lines marked in the 2D *x*–*y* overviews (white lines depict the axial extent of the marked granules with given values; white arrows in the 600 µm above and below the equatorial plane represent points of material release). Red-bordered inset in **i**: zoom-in of volume marked by the red box in the overview image with blue colors rendering the nucleus stain and blue-to-red depicting the granules with their respective color-coded normalized volume size (see color map label). Table in **e**: geometrical parameter values of granules marked by the respective numbers in the overview image with Vol = volume, Sur = surface, Len = longest axial length, and Wid = longest lateral axial width. Graph insets in the right panels: Intensity profiles along the white lines marked in the images with values giving the lengths of the lines in the profiles. Data are representative of three separate experiments

Confocal images of filaggrin-positive keratinocytes revealed diversity in the shapes and positions of the KHGs, both with respect to distance to the nucleus as well as to calcium concentration (Fig. 2). With increasing differentiation status, the primarily spherical or amorphous KHGs elongated and increasingly polarized, showing parallel alignment along the basal–apical axis of the cell (Fig. 2c, e, g, i). KHG size increased toward proximity of the nucleus (Fig. 2i, inset) where they often aggregated; this aggregation was the strongest at high calcium concentrations. Additionally, with increasing calcium levels, we noted the appearance of ring-like presentations of the larger KHGs and the formation of tube-like morphologies (Fig. 2c–h and Movie 1). The ring-like granules were primarily observed at the concentration of [Ca^2+^] = 2.5 mM in the proximity of the nucleus or within its limits (Fig. 2i and Figure S4B). Again, the coexistence of large, fully stained granules and granule aggregates (often in the same cell; Fig. 2i), the presence of tubular morphologies regardless of the small granule size (Fig. 2e, inset), and the presence of half-rings (Fig. 2e, inset in h and Movie 1; white arrows) excluded artifacts due to the incomplete antibody penetration during staining. The features we observed mirrored our previous findings in the uppermost epidermal layer. Finally, at the highest calcium level tested ([Ca^2+^] = 5 mM), KHGs disintegrated leaving little morphological definition; we could not detect tubular or ring-like formations. As a consequence, filaggrin staining at this calcium level was speckled and almost continuously distributed throughout the cytoplasm, i.e., the cell was completely filled with filaggrin^+^ material (Fig. 2k–l and Figure S4B), suggesting that the last stage of KHG maturation is fragmentation and filaggrin release. Since, as mentioned, the G-20 antibody is polyclonal and could potentially give some off-target staining, we have repeated these experiments with the N-terminal-specific, well-characterized anti-filaggrin antibody (15C10) (Figure S4C). The staining showed the same pattern, and we could observe the respective morphology and location changes of the KHGs. This, again, assured us that the antibody we used is, indeed, specific to the profilaggrin/filaggrin-containing granules and does not stain nonrelevant protein aggregates, such as aggregates of filaggrin-processing products or nonrelevant proteins.

**Fig. 3:**
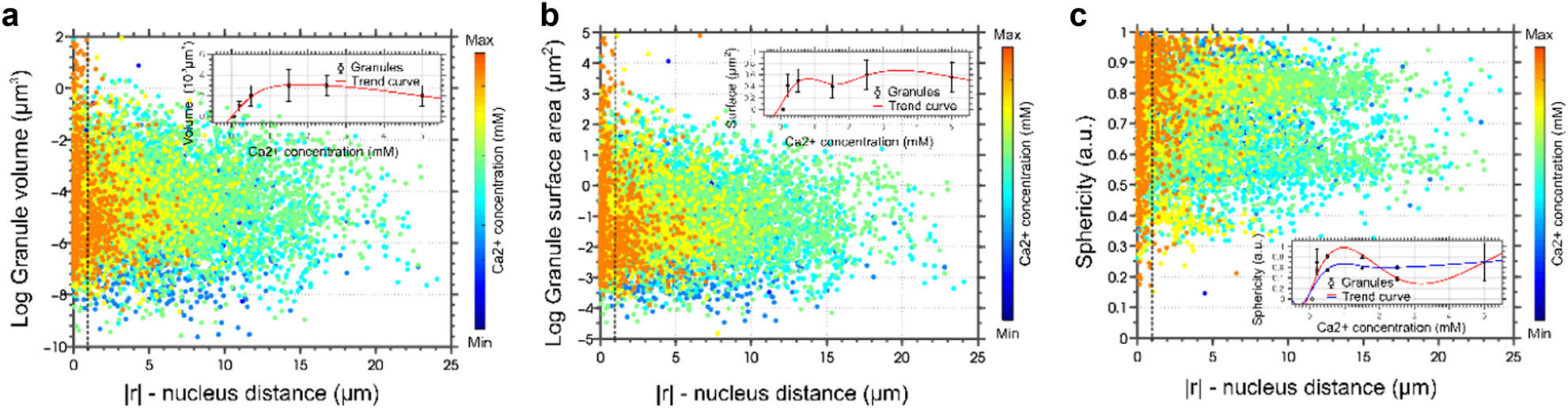
Filaggrin-containing granules undergo a shift in spatial distribution and locate in the proximity and within the limits of the cell nucleus. **a**–**c** Characterization of KHG population from confocal scanning microscopy data of fixed NHEK cells at different calcium switches, i.e., differentiation stages of Fig. 2. **a** Granule volume, **b** surface area, and **c** sphericity as a function of the distance to the nuclear membrane. Color coding: cold colors––low calcium = low differentiation state; warm colors––high calcium = high differentiation state (see also color bar). The dashed vertical line represents the reference point of all granule locations (nuclear membrane). The distance |r| is the absolute value of the differences of granule spatial coordinates; consequently, granules located within the nucleus had at least one negative spatial coordinate (*x*, *y*, or *z*). Insets: dependence of the average value of the respective parameter on calcium concentration [Ca^2+^]; >50 cells analyzed; error bars = SDM; *p* < 0.01, *n* = 11152 in ~50 cells analyzed; data are representative of three separate experiments. Red and blue lines represent fitted polynomial trend curves to the data. In **c**, average values are given for granules smaller than 3 µm in axial length (with red trend line) and for granules larger than 3 µm in length (with blue trend line)

Detailed analysis of the granules’ position (distance to the nucleus) and morphology (volume, surface area, and sphericity) in the 3D reconstructed images confirmed distinct correlations between both characteristics at different calcium levels (Figs. 2 and 3, and Figures S4B and S5). At low calcium levels, granules were evenly scattered within the cytoplasm. At higher calcium levels, KHG volume and surface area decreased consistently toward the periphery, leading to the accumulation of larger and more elongated granules near the nucleus. Further, we observed changes mirroring the findings in the epidermis; the concentration of KHGs and their geometric properties (length, surface area, volume, and sphericity) increased monotonically with calcium concentration until [Ca^2+^] = 1.5 mM, then plateaued, or decreased. To ensure that the changes in morphology and localization were not artifactual and could be observed in time and space, we carried out live imaging of NHEKs undergoing differentiation (Supplementary Text 1, Figure S6, and Movie 2–3).

### Actins form structures associating with filaggrin-containing KHGs

**Fig. 4:**
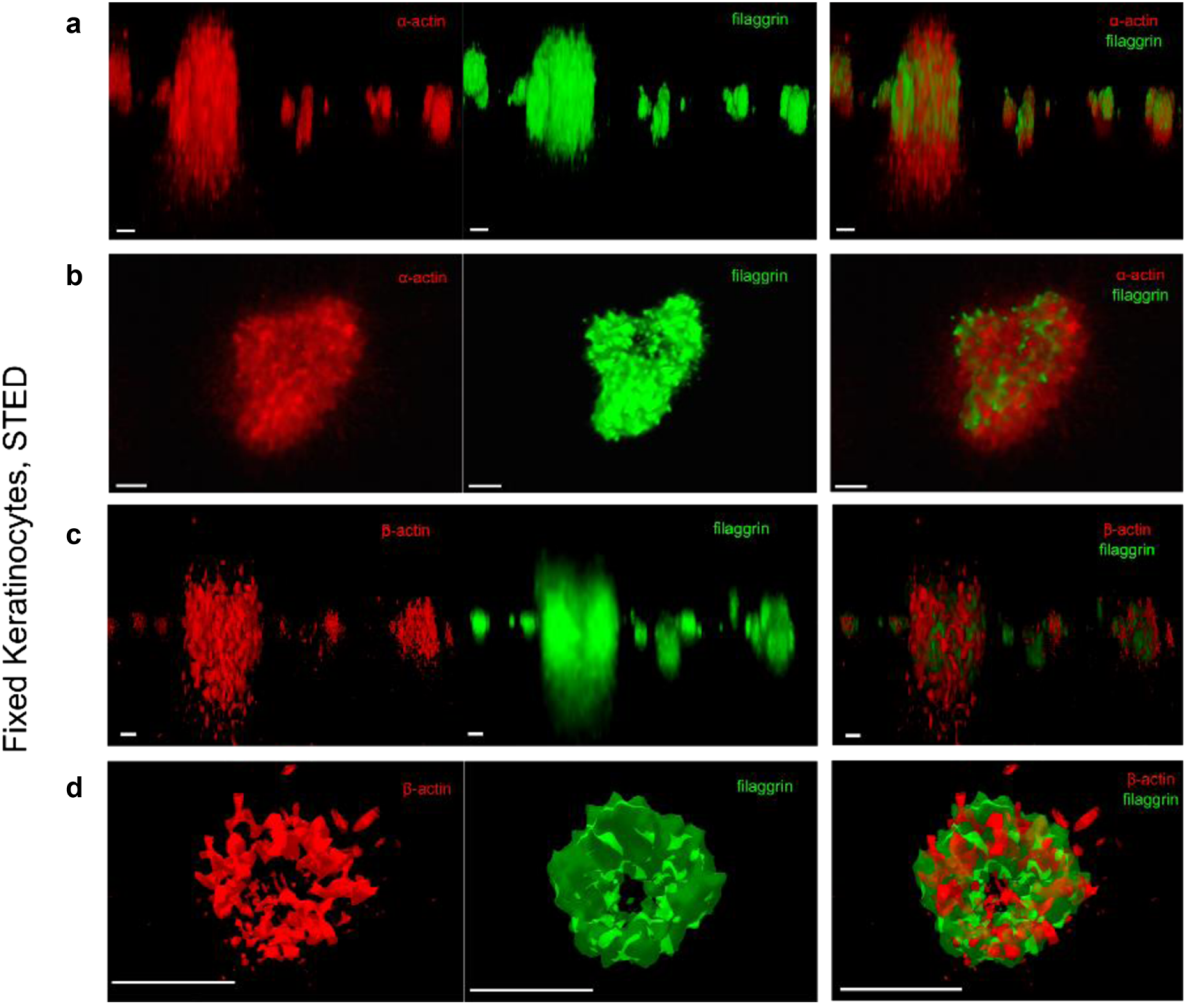
Actins form granule-associated scaffold structures. **a**–**d** Two-color 3D-STED images of fixed NHEKs 24 h after “calcium switch” to (Ca^2+^) = 1.5 mM, immunostaining for filaggrin (green, left panels; G-20 primary antibody, Alexa Fluor 488 as secondary antibody), and different actins (red; middle panels); right panels overlay of both. α-Actin (1A4 primary, secondary Alexa Fluor 568) and β-actin (4C2 primary, Alexa Fluor 568 secondary), side view (**a**, **c**) and top view (**b**,** d**). Scale bar 1 µm. Deconvolved images. Data are representative of three separate experiments

Since changes in the shape and location of organelles likely involve mechanical forces, we next investigated the actin cytoskeleton; the best known structure and the most probable candidate for intracellular force generation. We observed both natural collapse of F-actin during keratinocyte differentiation and the coinciding filaggrin and actin colocalizations (Supplementary Text 2 and Figures S7–S9); this prompted us to investigate the spatial organization between actin- and filaggrin-containing KHGs in more detail, by turning to super-resolution 3D-STED microscopy with increased axial (~300 nm) and lateral (~80 nm) resolution compared to confocal microscopes. Multicolor 3D-STED microscopy revealed the existence of actin-based KHG-associated scaffold structures ([Ca^2+^] = 1.5 mM) (Fig. 4 and Figure S10). We found differential roles for actin isoforms; α-actin shaped a central core (Fig. 4a, b), while β-actin appeared to form a cage-like structure surrounding the filaggrin^+^ material (Fig. 4c, d). Otherwise, abundantly expressed in keratinocytes as a part of the cytoplasmic actin network, γ-actin, appeared to be excluded from the scaffold structures (Figure S11); increased colocalization between this isoform and filaggrin at higher calcium concentration, and was therefore most likely due to increased γ-actin colocalization with material released to the cytoplasm and not KHGs themselves.

### Actin disruption increases filaggrin expression but disrupts granule integrity

**Fig. 5:**
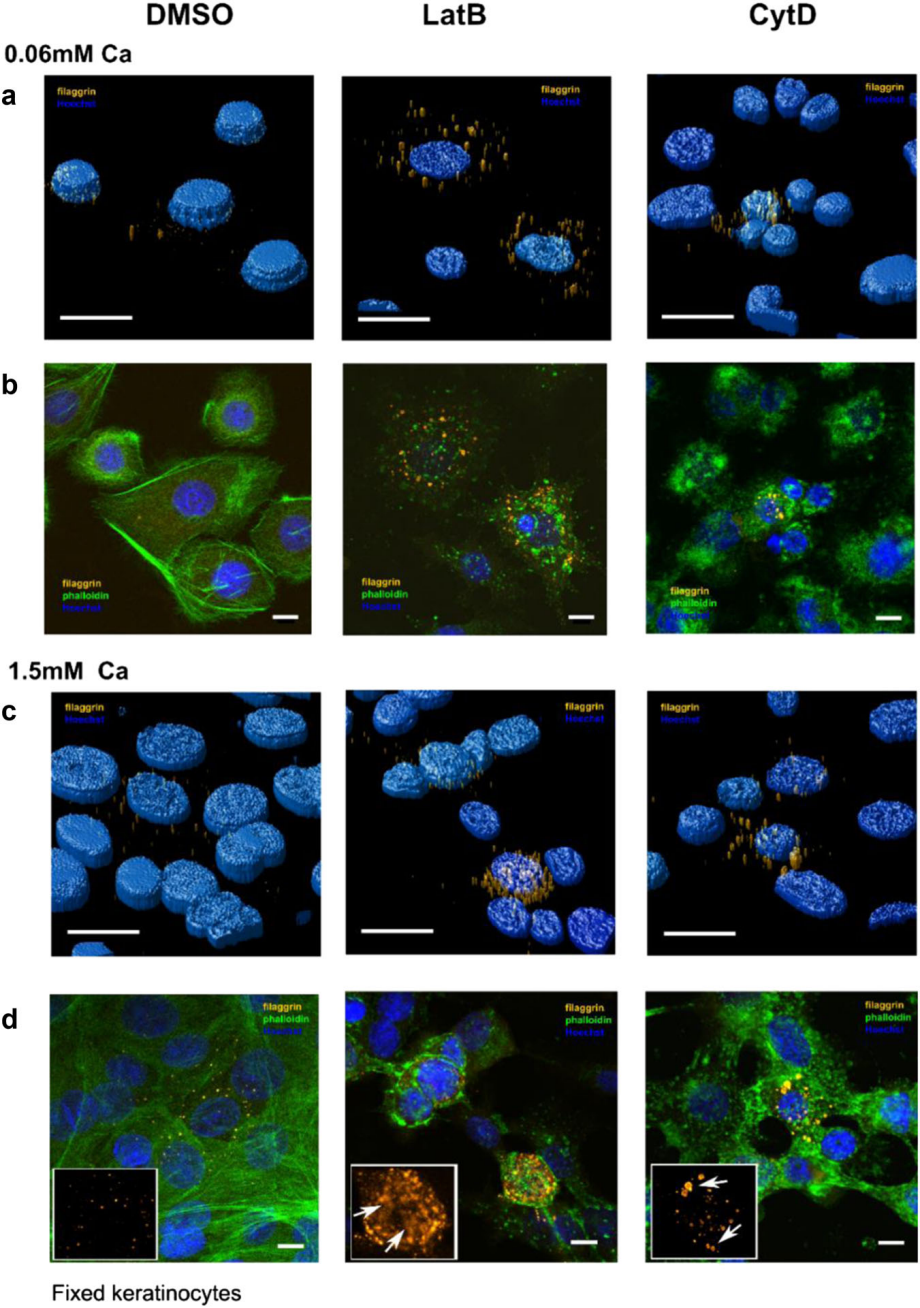
Actin inhibition increases filaggrin expression but disrupts granule integrity. Scanning confocal images of fixed NHEKs immunostained for filaggrin (orange, G-20 antibody and Alexa Fluor 488 secondary antibody), F-actin (green, phalloidin-488), and nuclei (blue, Hoechst) without treatment (left panels), with latrunculin B (LatB, middle panels), and with cytochalasin D (CytoD, right panels) treatment after 24 h of treatment with (**a**, **b**) [Ca^2+^] = 0.06 mM and (**c**, **d**) [Ca^2+^] = 1.5 mM. **a**, **c** 3D deconvolved images and **b**, **d** 2D single *X*–*Y* plane images. Insets in **d**: close-ups and arrows visualize intact granules (right panel) or filaggrin^+^ clouds (middle panel). Scale bars 10 µm. Data are representative of four separate experiments

To investigate the role of the scaffolds in KHG maturation, we next blocked actins pharmacologically with latrunculin B (LatB). LatB sequesters actin monomers, inhibiting F-actin polymerization in favor of F-actin depolymerization. The treatment resulted in a profound disruption of the F-actin network within few minutes (Figure S12). After 24 h, we observed changes in filaggrin staining and KHG formation (Fig. 5, left and middle panels). First, compared to untreated cells, both the abundance of the protein and formation of the “mature” tubular forms was dramatically increased already at low calcium concentrations [Ca^2+^] = 0.06 mM, suggesting accelerated differentiation in the presence of LatB. Higher calcium levels ([Ca^2+^] = 1.5 mM) combined with LatB treatment resulted in a dramatic increase in filaggrin expression and more filaggrin^+^ cells. Here, granules were smaller and surrounded by filaggrin^+^ material dispersed in the cytoplasm (Fig. 5d), as in terminally differentiated keratinocytes at [Ca^2+^] = 5 mM described above, i.e., the final stages of granule maturation (compare Fig. 2k and Figure S4B).

To further dissect this, we used cytochalasin D (CytD) (Fig. 5, right panels), which selectively inhibits short Arp2/3-complex nucleated filaments by blocking growing barbed ends of actin filaments, while having less-pronounced effects on long formin-mediated filaments^32,34^. We observed less-severe disruption of the cytoskeleton and differences in granule morphology compared with LatB treatment; KHGs expressed under CytD treatment were of the “intermediate” phenotype. At a low calcium level, granules were increased in number but relatively “immature” compared with the respective tubular phenotypes in LatB-treated cells (Fig. 5a, b, middle and right panels). In contrast, multiple “mature” tubular formations were present in CytD-treated keratinocytes at 1.5 mM calcium. Unlike in the corresponding LatB-treated cells, however, the integrity of these KHGs was preserved; we did not observe cytoplasmic filaggrin^+^ material. Similarly, we observed an intermediate level of phenotypic changes in cell morphology (Fig. 5).

Combined, these observations suggest that the shape and integrity of the KHGs are maintained predominantly by long formin-mediated actin filaments, while short Arp2/3-mediated filaments are required to prevent the escape of filaggrin^+^ material, resulting in N-terminal domain signaling, leading to premature keratinocyte differentiation and death.

### AKT1 mediates a switch between actin stabilization and filaggrin processing

**Fig. 6:**
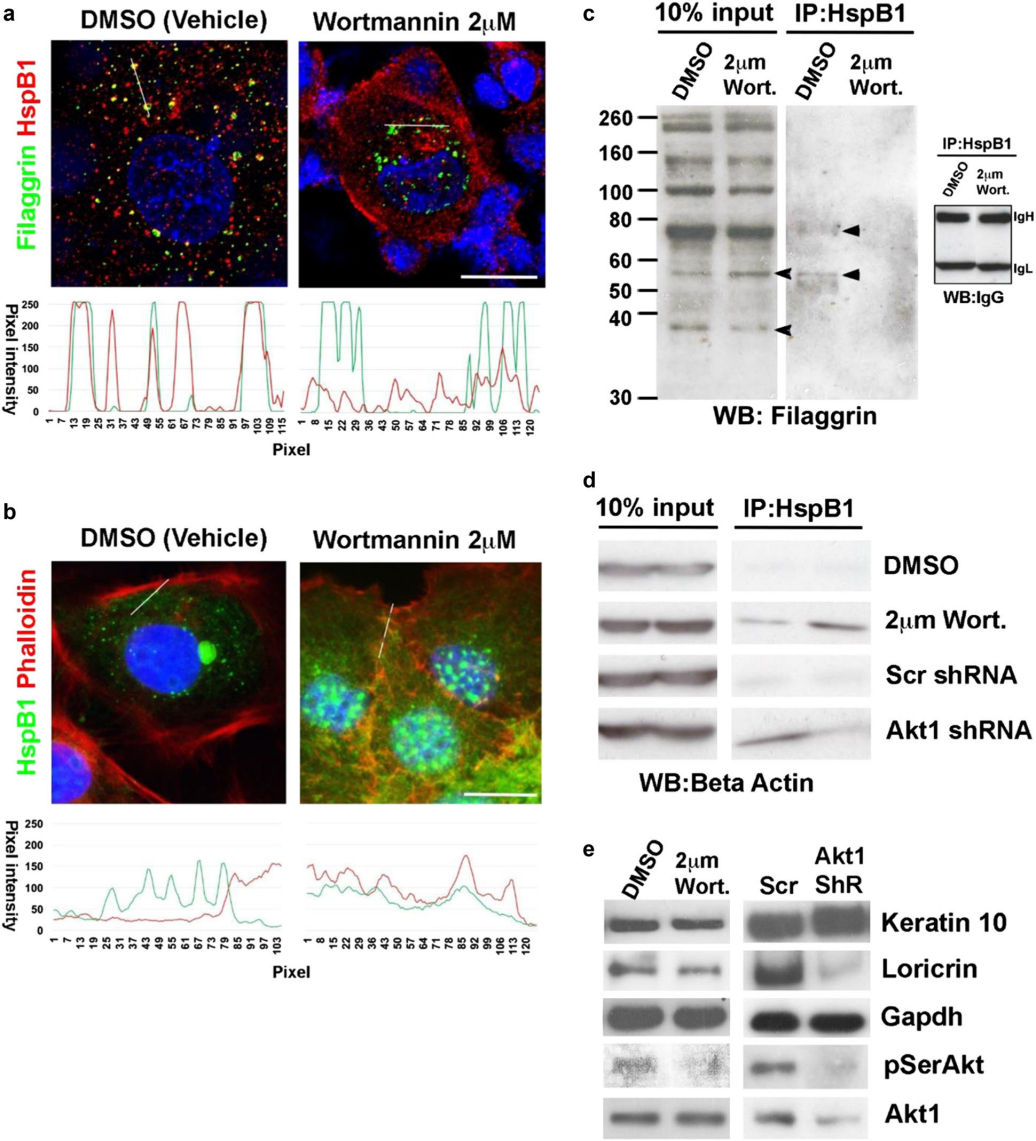
AKT1-dependent switch between HspB1 interaction with actin and HspB1 interaction with filaggrin and filaggrin processing. **a**, **b** Confocal microscopy of fixed postconfluent rat epidermal keratinocytes treated with vehicle (DMSO) or 2 μm of wortmannin and stained for nucleus (blue, DAPI) and **a** filaggrin (green, Alexa Fluor 488) and HspB1 (red, Alexa Fluor 594) or **b** HspB1 (green, Alexa Fluor 488) and actin (red, TRITC–phalloidin). The graphs in each figure show pixel intensity for the red and green channels, respectively, along the line indicated in the micrographs. Scale bar 10 μm. **c**, **d** Co-immunoprecipitation of **c** HspB1 and filaggrin and **d** HspB1 and β-actin in postconfluent rat epidermal keratinocytes treated with DMSO or wortmannin (Wort); or expressing scrambled or Akt1 shRNA as labeled. 10% Input shows total **c** filaggrin or **d** actin (data represent biological replicates: two separate IP experiments). IgH and IgL are IgG heavy and light chains which serve as a loading control for the immunoprecipitation. Arrowheads show the processed filaggrin species altered by wortmannin treatment, and the filaggrin intermediates that are immunoprecipitated by HspB1. **e** Western blot of keratin-10, loricrin pSer 473 Akt, and AKT1, in wortmannin treated or AKT1 shRNA knockdown keratinocyte (Akt1 ShR, *n* = 3). GAPDH serves as a loading control

Next, we investigated whether AKT1 and HspB1 played a role in actin disruption during epidermal terminal differentiation. As mentioned, HspB1 binds and stabilizes actin^36,37^. It further specifically binds to filaggrin in an AKT1-dependent manner during epidermal terminal differentiation to facilitate filaggrin processing, and is present in the KHGs of differentiated keratinocytes^39^. We therefore treated rat epidermal keratinocytes with wortmannin, which is known to inhibit AKT1 in keratinocytes^44^; colocalization between filaggrin and HspB1 in granules was prevented with wortmannin treatment (Fig. 6a). In untreated cells, actin was predominantly cortical, with only small amounts of actin colocalizing with HspB1 in the cytosol, while it extensively colocalized with HspB1 upon wortmannin treatment (Fig. 6b). Expression of the processed form of filaggrin was also altered, as was interaction between the two proteins measured by immunoprecipitation (Fig. 6c). Immunoprecipitation experiments highlighted an increased interaction between HspB1 and β-actin upon conditions reducing AKT1 function, i.e., both in wortmannin-treated keratinocytes and those expressing AKT1 shRNA (Fig. 6d). Wortmannin treatment was similar to shRNA knockdown in that it reduced both expression of a differentiation marker loricrin and phosphorylated AKT (pSerAKT) (Fig. 6e).

### Actin disruption leads to accelerated cornification

**Fig. 7:**
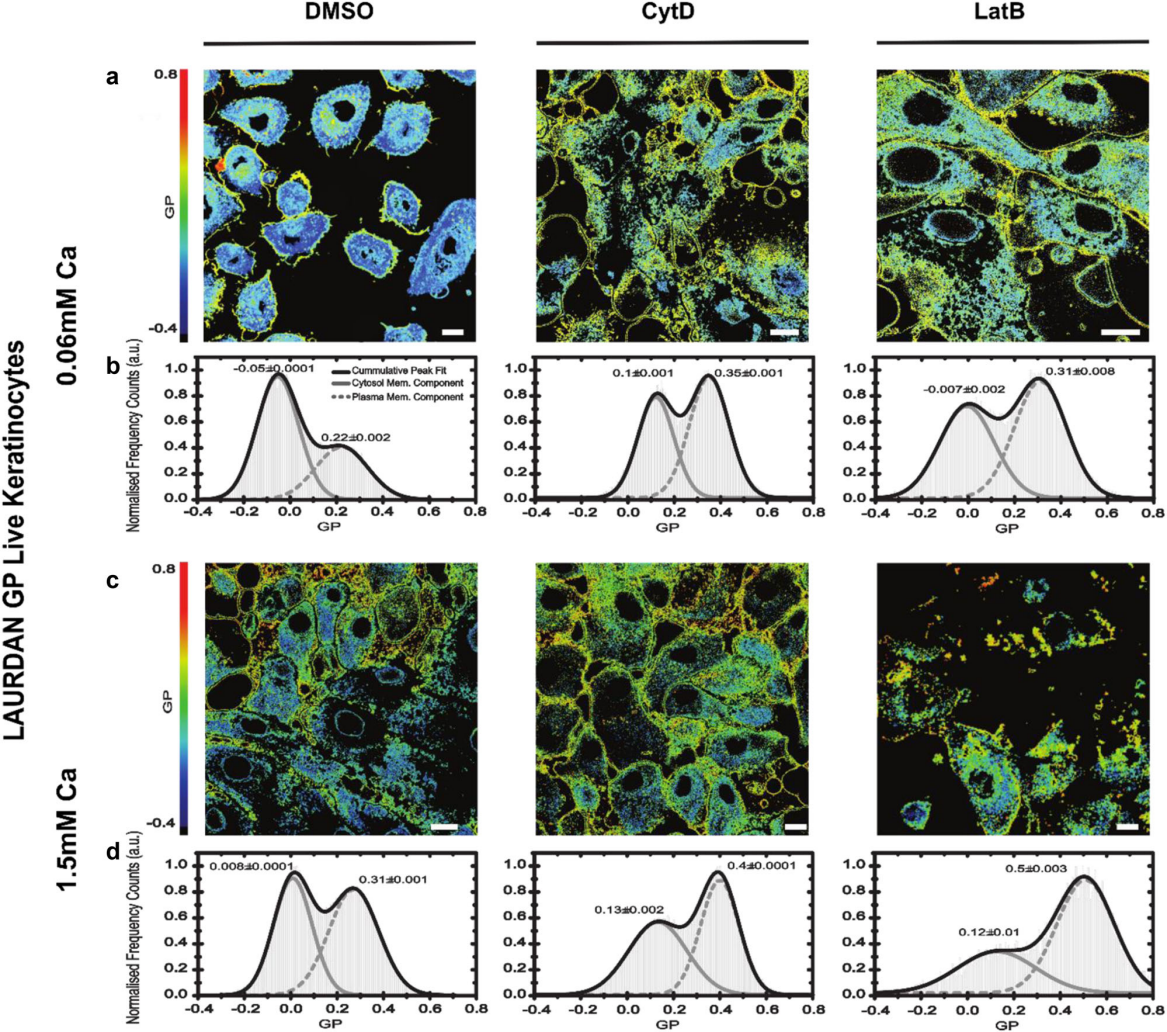
Actin disruption leads to accelerated cornification. **a**–**d** GP images (**a**,** c**) and histograms of GP values over all image pixels generated from 2D scanning confocal images of live NHEKs labeled with the polarity-sensitive lipid analog dye Laurdan; large GP values indicate increased stiffness of the cellular membranes. Conditions without any treatment (left panels) and with cytochalasin D (CytD, middle panels) and latrunculin B (LatB, right panels) treatment after treatment at low [Ca^2+^] = 0.6 mM (**a**, **b**) and high [Ca^2+^] = 1.5 mM (**c**, **d**) calcium levels. Scale bar 10 µm. Data are representative of three separate experiments, at least *n* = 15 cells per condition. The left (lower values) and right (higher values) peaks in the GP histograms indicate the cytosolic and membrane components, respectively, highlighting an increase in membrane stiffness for larger calcium levels, i.e., increased differentiation, and after actin disruption

Finally, we explored how changes in actin affect cornification. Cornification involves the formation of a rigid “cornified envelope” and membrane bilayer remodeling^3,45–47^, resulting in the increased stiffness of the plasma membrane. While there are of course certain limitations of the model when using an in vitro monolayer, terminal differentiation of keratinocytes (including processes considered as a part of “cornification”) can be readily observed in 2D cultures. Hence, we next quantified the fluidity of the plasma membrane and thus stiffness in live NHEKs using LAURDAN (6-dodecanoyl-2-dimethylaminonaphthalene), a polarity-sensitive lipid analog dye. In LAURDAN imaging, membrane stiffness is reported as “general polarization”^48–50^ (GP; higher values indicate stiffer membranes). We have developed an approach to study the advancement of differentiation in live keratinocytes. With this, we found a positive correlation between calcium concentration (0.06–5 mM) and the membrane stiffness. GP values increased more than expected following treatment with actin inhibitors (Fig. 7), mirroring our previous findings on KHGs and cell morphology. Again, we observed differences between treatments; LatB induced a more pronounced increase in GP values (stiffer membranes) than CytD, with plasma membranes stiff already at levels of 1.5 mM calcium, corresponding to the phenotype of a cell progressing through the cornification process.

Collectively, these findings show that actin disruption accelerates programs of keratinocyte differentiation and implicates both long and short F-actin filaments in preventing premature cornification.

## Discussion

**Fig. 8:**
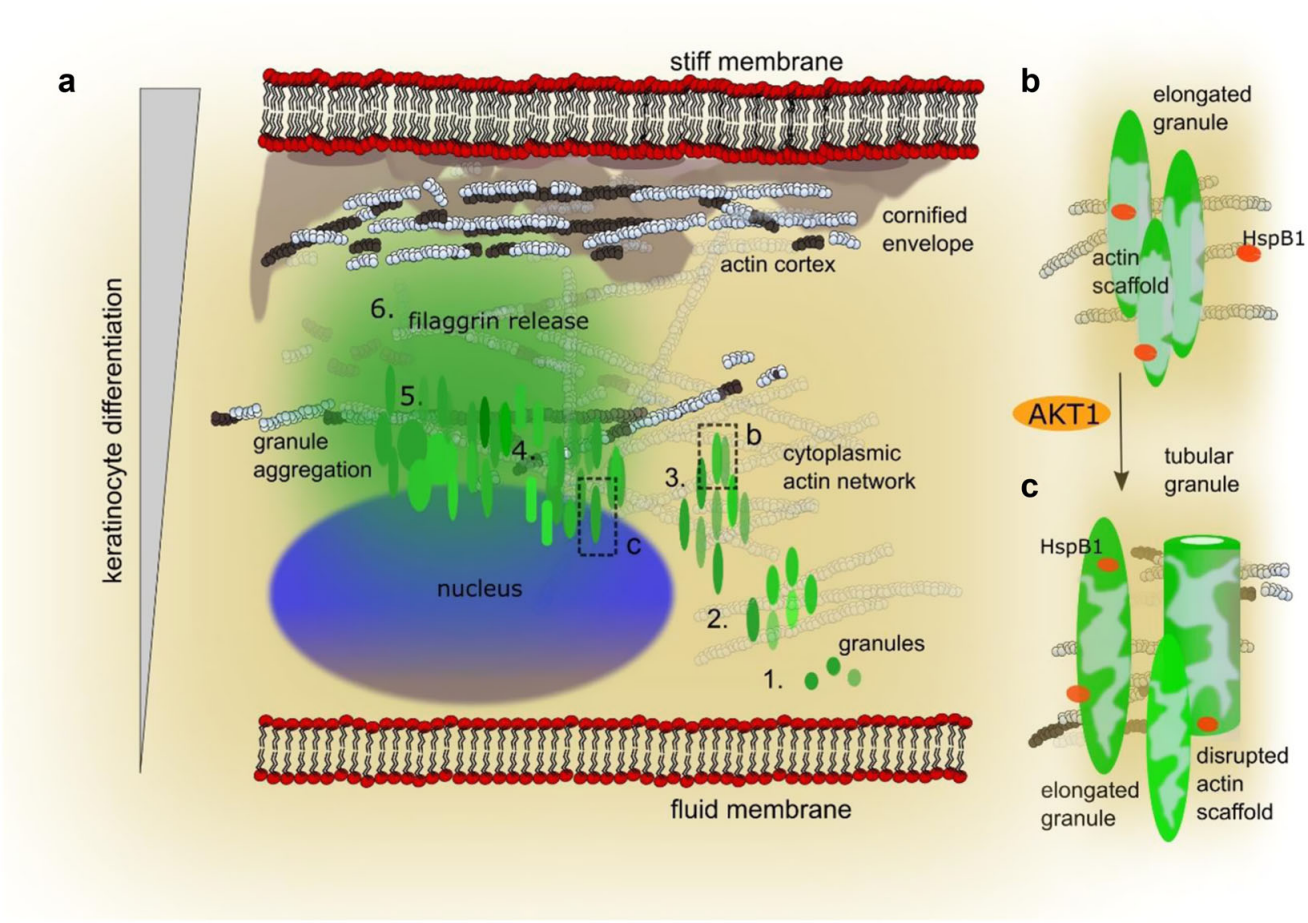
Proposed model of filaggrin and actin dynamics during keratinocyte differentiation and cornification. **a** Different stages of “keratohyalin granule (KHG) maturation” and actin organization within keratinocytes with plasma membrane (labeled “membrane” with lipid chains, black, and headgroups, red dots), nucleus (blue), granules (green), actin (1–3 and **b**: white represents intact filaments; 4–6 and **c**: white and black represent compromised filaments), cornified envelope (gray shaded area) vs. increased differentiation, and cornification from bottom to top (left gray bar): 1) intense profilaggrin expression and local precipitation in the form of nascent KHGs; 2) granule association with the actin network; 3) granule morphology changes—shape elongation and increase in surface area facilitating the access of processing enzymes for intensified monomer generation; 4) localization shift toward the nucleus to further enhance pro-differentiation signaling and increase in the shape complexity; 5) expression of profilaggrin processing enzymes and intensified generation of filaggrin monomers, interaction of monomers with the granule-associated actin lattice, and actin cytoskeleton collapse; and 6) rapid intracellular release of filaggrin from KHGs, binding of the free filaggrin monomers to keratin-based intermediate filaments, leading to the collapse of the IF network; stabilization of cornified envelope (CE). (**b**,** c**) AKT1-mediated switch between HspB1/actin and HspB1/filaggrin binding. Zoom-ins into stages marked by black dotted boxes in a: granule elongation (**b**, stage 3), and mature granules with disrupted actin scaffold (**c**, stage 4); light-green areas schematically mark the integrity of granule-associated actin scaffold

While filaggrin insufficiency is strongly associated with multiple inflammatory diseases, relatively little is known about filaggrin homeostasis and function. Using advanced microscopy on both human epidermal tissue and cultured keratinocytes, we show novel details on the structural organization and dynamics of filaggrin-containing KHGs during epidermal differentiation and cornification. Our study suggests an extended model of cornification where the controlling mechanism for keratinocyte differentiation and death involves actins, supporting both filaggrin storage integrity and initial sequestration within KHGs, and rapid filaggrin release, enabling the formation of a fully functional skin barrier (Fig. 8). The actin-based cytoskeleton may offer additional advantages for the control of KHG maturation, e.g., promoting perinuclear localization for nuclear expulsion^27^ and/or filaggrin N-terminal effects on gene expression^28^. Actin scaffolds may also help prevent consequences of premature filaggrin escape^51^ from the granules into the cytoplasm (Fig. 8a, stages 1–3 and b); and the eventual cytoskeleton collapse^52,53^ (Fig. 8a, stages 4–5 and c). Therefore, it likely provides an “on-switch” supporting cytoplasmic accumulation of filaggrin monomers (Fig. 8a, stage 6), to trigger cell death and *stratum corneum* formation. While there is substantial evidence of the association of filaggrin and KHGs with keratin filaments^54^, which participate in granule formation (reviewed in ref.^55^), keratins do not offer such a mechanism to control filaggrin release.

Possible scenarios to explain the effects of actin inhibition on KHGs and cornification may include either cytoskeleton disruption, granule disintegration, or the interplay of both. Since the effects were not immediate, they are unlikely to occur due to the disruption of actin filaments per se, as disassembly signals affect F-actin organization on a short time scale. Instead, these could be a direct result of material release from KHGs and translocation of the N-terminal domain to the nucleus for subsequent activation of pro-differentiation signals^27,28^. Indeed, the observed unrestricted filaggrin expression and consequent cornification acceleration following actin inhibition would support this.

Mechanistically, it is likely that both containment of granule material and shape/morphology change may require participation of either short Arp2/3-nucleated and/or long formin-mediated filaments. Therefore, we used LatB and CytD, to either generally facilitate F-actin depolymerization or to selectively inhibit the short Arp2/3-nucleated filaments, respectively. Albeit with the limitations of keratinocyte monolayers, while we also observed morphological changes in KHGs, they were less pronounced in the presence of CytD, indicating that the actin scaffolds were to a large extent maintained by the formin-mediated long actin filaments rather than Arp2/3-mediated F-actin; while short filaments may prevent excessive leakage of material from the granules.

In addition to constituting a core cytoskeleton, actins have been implicated in multiple roles in the epidermis, controlling motility, stratification, wound healing, and stem cell maintenance^53,56–58^, as well as responses following barrier disruption^59^. The varied expression pattern of actin isoforms in keratinocytes at subsequent differentiation stages suggests their importance during cornification. Currently, we do not yet understand the abundance of γ1-actin, forming the majority of the cytoplasmic actin network in keratinocytes. However, involvement of the actin skeleton in organelle movement has been well established (reviewed by Cramer^60^), hence it could potentially contribute to the observed KHG shift toward the nucleus.

Multiple actin/actin family genes localize within or near chromosomal regions identified in linkage and genome-wide association studies (GWAS) of AD and psoriasis, including shared loci^61–65^. Interestingly, deficiency in the actin system results in skin abnormalities and linked allergic/atopic phenotypes. Specifically, Wiskott–Aldrich syndrome patients, in whom a defect in WASp (Wiskott–Aldrich syndrome protein) has been identified, present with a severe and difficult-to-treat dermatitis and allergic manifestations. However, X-linked thrombocytopenia patients, carrying a different mutation in WASp, tend to present with milder or no dermatitis^66^. WASp and related proteins act as important actin reorganization and polymerization activators^67^; it has been previously proposed that the mutation in the *WAS* gene could contribute to AD^73^. The exact role of α- and β-actins in the KHG scaffolds remains unclear because they could not be selectively inhibited with chemical inhibitors. However, we show that the actin-stabilizing protein HspB1 is a potential mediator of (β-)actin disruption and filaggrin processing during keratinocyte differentiation by switching the binding partner from actin to filaggrin, dependent on the AKT1 status^39^. Future research should focus on systematic protein deletion to elucidate the molecular interactions and dynamics of different actins with profilaggrin/filaggrin at the molecular level.

In summary, we identified a novel, actin/HspB1/AKT1-dependent mechanism of filaggrin release from KHGs, acting as a switch between differentiation and cell death during skin barrier formation. Our results show that KHG morphology and dynamics are more complex than those previously considered; the mechanism identified may sequentially support both the development of fully functional stratified live epidermis and drive events, leading to cell death during the formation of *stratum corneum*. By addressing the structural and functional relationships of filaggrin with accessory molecules (including actins), our study suggests possible directions for the development of diagnostics and personalized treatment strategies for patients with filaggrin-associated inflammatory diseases and allergy.

## Materials and methods

### Human keratinocyte culture and calcium switch

Normal human epidermal keratinocytes (NHEKs) were purchased from Lonza (Lonza, Basel, Switzerland, neonatal, pooled). The cells were cultured in KBM-2 media (Lonza, Basel, Switzerland) at the calcium level of 0.06 mM and subcultured by Accutase (Sigma-Aldrich, Gillingham, Dorset, UK). Calcium switch was conducted over a period of 24 h by replacing the culture media with media adjusted to the desired calcium concentration by CaCl_2_.

### Rat keratinocyte culture

Aliquots of single-cell suspensions were transferred to culture plates or flasks with modified DMEM (DMEM+) and placed in the incubator. The media was replaced with fresh DMEM (Sigma-Aldrich, Gillingham, Dorset, UK) every 2 or 3 days, and cells were subcultured before they reach 90% confluency. For transfected cell lines, the cells were selected using DMEM supplemented with G418 (Sigma-Aldrich, Gillingham, Dorset, UK) at a concentration of 0.1 mg/ml as this is the concentration that prevented REK cell growth in assays. Cells were collected after washing the flasks or plates three times with PBS, followed by trypsinization with 0.25% trypsin (Sigma-Aldrich, Gillingham, Dorset, UK) for 5 min, and pelleted after centrifugation at 1600 × *g* for 20 min at room temperature.

### Epidermal sheet isolation

Skin samples were obtained from healthy donors undergoing surgery under ethical approval from the UK National Research Ethics Service (14.NW.1153). Epidermal sheets were isolated by overnight incubation in dispase (5 U/ml; Sigma-Aldrich, Gillingham, Dorset, UK) and separation of the epidermis from dermal tissues. For fluorescent antibody staining, these epidermal sheets were first incubated with 4% formaldehyde (Sigma-Aldrich, Gillingham, Dorset, UK), followed by 0.1% Triton X-100 (Sigma-Aldrich, Gillingham, Dorset, UK). The procedure followed the “Fluorescent antibody staining” protocol given below from the blocking stage.

### Fluorescent antibody staining

NHEKs were grown and subjected to the required treatment in eight-well cell culture slides (Beckton Dickinson), fixed and permeabilized by neat acetone, and incubated in blocking buffer (5% FCS, Sigma-Aldrich, Gillingham, Dorset, UK; 2% BSA, Sigma-Aldrich, Gillingham, Dorset, UK in PBS; or 0.4% fish skin gelatin dissolved in TBS and 0.2% Triton X-100). Filaggrin antibody (mouse 15C10 from Leica, Milton Keynes, UK; rabbit H-300, goat G-20 from Santa Cruz Biotechnology, Dallas, TX, USA, or FLG01 monoclonal from Genetex, Irvine, CA, USA in Fig. 6), keratin-10 (rabbit, Abcam, Cambridge, UK), α-, β- and γ-actin (1A4, 4C2, and 2A3, respectively; all mouse from Abcam, Cambridge, UK), HspB1 (rabbit, Abcam, Cambridge, UK) fluorescent phalloidin-Alexa 488 (Life Technologies/ThermoFisher Scientific, Waltham, MA, USA), and TRITC–phalloidin (Sigma-Aldrich, Gillingham, Dorset, UK) and the secondary antibodies (anti-mouse Alexa 488, anti-mouse Alexa568, anti-goat Alexa 488, and anti-rabbit Alexa568; all from Life Technologies/ThermoFisher Scientific, Waltham, MA, USA) staining was carried out in PBS, and nuclei were visualized by Hoechst (NucBlue, Life Technologies/ThermoFisher Scientific, Waltham, MA, USA) or DAPI. The cells on microscope cover-slides were mounted with Mowiol-488 (Sigma-Aldrich, Gillingham, Dorset, UK) or in Prolong Gold anti-fade reagent (Life Technologies/ThermoFisher Scientific, Waltham, MA, USA). Data acquisition was carried out on the Zeiss 780, Zeiss LSM 710 inverted confocal microscope (Zeiss, Jena, Germany), Nikon Eclipse E600 (Nikon, Tokyo, Japan), or Leica SP8 (Leica, Wetzlar, Germany) inverted confocal microscope by recording 2D images in different axial (3D) planes.

### Western blot and immunoprecipitation

Isolated epidermal sheets were incubated in 8 M urea buffer (ReadyPrep Sequential Extraction kit, Reagent 2 plus reducing reagent; Bio-Rad, Hercules, CA, USA) and sonicated in a water bath for 30 min. Lysates were spun at 4 °C (13,000 rpm, 15 min) and run on 7% TA gels (Life Technologies/ThermoFisher Scientific, Waltham, MA, USA) in Xcell SureLock Mini-Cell Electrophoresis System (Life Technologies/ThermoFisher Scientific, Waltham, MA, USA). Proteins were transferred onto PVDF membranes (iBlot Dry Blot system stacks and iBlot transfer device; Life Technologies/ThermoFisher Scientific, Waltham, MA, USA). Membranes were incubated in 5% solution of nonfat milk powder (Sigma-Aldrich, Gillingham, Dorset, UK) in PBS and then with the desired antibodies overnight. Li-Cor secondary antibodies and Li-Cor scanning system (Li-Cor Biosciences, Lincoln, NE, USA) were used for detection.

Immunoprecipitation (IP) was performed in keratinocytes lysed with ice freshly made cold-radioimmunoprecipitation buffer (RIPA) as previously described^39^. Lysates were incubated for 4 h at 4 °C in 1/25 v/v agarose conjugated with goat anti-HspB1 antibody (Santa Cruz Biotechnology, Dallas, Texas, USA). The following antibodies were used at the following concentrations: mouse anti-beta-actin 1:2000 (clone AC13; Sigma-Aldrich, Gillingham, Dorset, UK), and rabbit anti-filaggrin 1:500 (sc-30230 (M-290), Santa Cruz Biotechnology Biotechnology, Dallas, TX, USA).

### Live cell staining and observation of keratinocyte differentiation in live cells

In order to stain live cells intracellularly while maintaining plasma membrane integrity, we adapted a cationic lipid-aided intracellular staining protocol described by Weill et al.^68^. A mix of either primary anti-filaggrin (G-20; Santa Cruz Biotechnology, Dallas, TX, USA) or isotype control (goat; BD, Franklin Lakes, NJ, USA) antibody and secondary antibody (anti-goat Alexa 488; Life Technologies/ThermoFisher Scientific, Waltham, MA, USA) was prepared in low calcium ([Ca^2+^] = 0.06 mM) keratinocyte medium (KBM-2; Lonza) at 1:200 dilution. Lipofectamine 2000 cationic lipid reagent (Life Technologies/ThermoFisher Scientific, Waltham, MA, USA) was added at 0.5 µl/ml and incubated for 15 min. The cell culture medium was replaced by Lipofectamine 2000 containing a mix for 2–4 h and then washed off multiple times. The cell nuclei were stained using Hoechst (NucBlue Live Ready Probes, Life Technologies/ThermoFisher Scientific, Waltham, MA, USA). Calcium-supplemented KBM-2 medium (Lonza, Basel, Switzerland) ([Ca^2+^] = 1.5 mM or 5 mM) was added, and imaging (one Z-stack every hour; distance between each axial layer with a Z-stack: 300 nm) was carried out on the Leica SP8 confocal microscope (Leica Microsystems, Wetzlar, Germany) over a period of 24–48 h following the first calcium switch, with temperature and humidity control.

### AKT1 knockdown

Four SureSilencing shRNA plasmids (Qiagen, Paisley, UK) were used to knock down Akt1 expression in REKs; shRNA1-GCA CCG CTT CTT TGC CAA CAT, shRNA2-AAG GCA CAG GTC GCT ACT AT, shRNA3-GAG GCC CAA CAC CTT CAT CAT, and shRNA4-GCT GTT CGA GCT CAT CCT AAT, and of these, 1 and 3 were used for further experiments.

### AKT1 inhibition and actin perturbation experiments

Wortmannin (Sigma-Aldrich, Gillingham, Dorset, UK), a PI3 kinase inhibitor, was introduced into REK cells at 2 μM 24 h^S1^ prior to lysate collection. CytD and LatB were purchased from either Merck Biosciences, Darmstadt, Germany, or Abcam, Cambridge, UK. Drugs were added to the culture medium at the concentration of 5 µM and 2 µM, respectively, and the cells were incubated for variable amounts of time (between 1 min and 24 h); the inhibitors were washed out from the cultures at the end of culturing time and cells were fixed.

### 3D super-resolution STED imaging

The structural role of the different actin monomers and the tight connection with the filaggrin granules was studied employing 3D super-resolution STED microscopy. For this purpose, the NHEK cell monolayers at [Ca^2+^] = 1.5 mM were immunolabeled and fixed in a similar manner as described before with filaggrin (goat G-20 and anti-goat Alexa Fluor 488 secondary antibody, Life Technologies/ThermoFisher Scientific, Waltham, MA, USA) and α-, β-, and γ-actin (1A4, 4C2, and 2A3, respectively, and anti-mouse Alexa Fluor 568, Life Technologies/ThermoFisher Scientific, Waltham, MA, USA). STED microscopy was carried out on a Leica TCS SP8 3× gated STED (Leica, Wetzlar, Germany), equipped with a pulsed supercontinuum white-light excitation laser at 80 MHz (NKT, Copenhagen, Denmark), and two continuous-wavelength STED lasers at 592 nm and 660 nm. Experiments with filaggrin_488 and actin_568 were excited at 488 nm, their emission was depleted at 592 and 660 nm detected (employing Leica HyD detectors in gating mode, time gate for detection was 1.5–6 ns) around 530 nm and 600 nm, respectively. For 3D-STED imaging, the depletion lasers were split into two different optical paths, each one with an independent phase plate to form the doughnut-shaped pattern along the lateral (*xy*) and axial (*z*) direction. By means of a variable beam splitter, a relative percentage of STED laser power was sent either into the beam paths for lateral or axial confinement (in our case 50:50), making possible the increase of resolution in the three dimensions. A sequential imaging mode was set to obtain in the first instance the super-resolved images of each actin monomer employing the 660-nm STED laser, and later the 592-nm STED laser for the filaggrin. The 3D obtained images were surface rendered employing the 3D imaging Leica software. The raw images shown in Figure S10 were imported into ImageJ, and subsequently exported as tiff; a set of a minimum of three images were taken from three different cellular batches.

### Granule data analysis, plotting, and image representation

We employed a multistage data-processing protocol. Specifically, we implemented several steps of image analysis for accurate granule localization, quantification and characterization, plotting, and image representation, using several image acquisition, analysis, and graphing software. Briefly, unless stated otherwise; (1) every single confocal plane of a 3D imaging stack was first deconvolved employing the Huygens Professional software package (Huygens; Scientific Volume Imaging, Hilversum, the Netherlands); (2) the resulting 3D image was surface rendered (Huygens), and the 3D isosurface was used for quantitative analysis; (3) a custom-built macro within the advanced object analysis module of Huygens was used to localize, quantify, and characterize the geometry of each individual granule; and (4) the resulting data matrices were exported into graphing and statistics software, such as Origin Pro (OriginLab Corporation, Northampton, MA, USA), MATLAB (Mathworks, Natick, MA, USA), and Prism 6.04 (GraphPad Software, La Jolla, CA, USA) for plotting.

The representation of the 2D or 3D images of the epidermis, cells, and granules isosurfaces was realized using Huygens Professional. The LAX software (Leica SP8, Leica, Wetzlar, Germany) was employed to generate the surface-rendered 3D-STED images, the granule 3D video reconstruction, and the 3D raw images. Fiji (ImageJ; NIH, US) was used to present the raw 2D confocal images.

#### Image deconvolution

Reduction of image artifacts due to noise and optical aberrations (possibly resulting in a reduced accuracy to localize and geometrically characterize the granules) was minimized by deconvolution (Huygens Professional; for details, see https://svi.nl/HuygensDeconvolution). For deconvolution of the 2D images, we generated a custom point-spread function by imaging fluorescent beads (Figure S3). A theoretical point-spread function defined by the software (adjusted as a function of the penetration height/depth) was employed for deconvolution of the 3D images.

#### Granule localization and quantitative analysis of geometrical parameters

To accurately assess the single-cell individual localization of the granules in 3D, we used the advanced object characterization module of the Huygens software package to identify the center of mass of each granule within the 3D reconstructed images. The resulting *x*, *y*, and *z* coordinates were used to obtain the relative distances of the KHGs to the nucleus surface and their geometric values. For the latter, an isosurface was defined as the 3D surface representation of points with equal fluorescence intensity values; the geometric parameter “granule volume” measured the total volume of the isosurface of granules; the geometric parameter “surface area” measured the total area of the isosurface of granules; and the parameter granule “length” measured the largest distance along the three principal axes of a box enclosing the 3D isovolume granule. Finally, the sphericity (Sph) parameter, defined as the roughness of the 3D isosurface of the granules, is a measure of how close the volume-to-surface ratio was to that of an ideal sphere

$${\rm{Sph}} = \pi ^{1/3} \left[ {\frac{{6 \cdot V_{\rm{iso}}}}{{A_{\rm{iso}}}}} \right]^{2/3},$$

where *V*_iso_ is the 3D isovolume and *A*_iso_ is the 3D isosurface of the segmented object. For graphical representation of the distribution of the geometrical granule parameters, a custom-written MATLAB batch mode was designed to read the parameter values along with the granule’s axial *z* localization, normalize the latter to the total height of the respective epidermal sample (sample number *n* = 3), and then concatenate the parameter matrices for all the individual epidermal sample files. The distributions of parameter values were represented in scatter-plots (e.g., Figure 1 and E2). Note, Huygens employs a minimum sampling limit accounting for the lateral *x*, *y*, and axial *z* spatial resolution of the confocal microscope which is limited to about 200 nm and 800 nm, respectively, due to diffraction. Consequently, structures with vertical spacing below these values cannot be resolved accurately, and the scatter plot of the geometric parameter “length” shows discrete patterning. We can exclude optical aberrations due to imaging depth as a reason of the geometrical changes, since the deepest layers were closest to the microscope cover glass (Fig. 1a). The same analysis procedure was also used to define the position of the granules with respect to the nucleus in keratinocytes. Custom-built software algorithm was designed to determine the distance of each granule to the nucleus in the same cell. For this, the *x*, *y*, and *z* localization of each granule was related to the isosurface of the nucleus. The isosurface of the nucleus was defined as the 3D surface representation of points with equal fluorescence intensity with coordinates (*x*,*y*,*z*). Granules resulting in a negative distance in *x* and/or *y* coordinate were considered to be inside and those with positive distances along *x* and *y* outside of the nucleus. In addition to the previously defined four geometric parameters, in the keratinocytes, we also evaluated the axial and lateral width of the granules, since these parameters provided a more sensitive analysis with respect to changes in granule shape upon keratinocyte differentiation. The axial aspect ratio “AxRatio” is the ratio of the object length to its axial width, and the lateral aspect ratio “LaRatio” is the ratio of the object length to its axial width. The values of these parameters were calculated using a custom-built macro within the advanced object characterization module of the Huygens software package, and further transferred to a custom-written MATLAB batch routine that concatenated and translated them into parameter matrices at each calcium concentration. A scatter plot was generated depicting the granule geometric parameters as a function of their distance to the nucleus surface, with cold to warm colors according to low and high calcium concentrations, respectively (Fig. 3 and Figure S5). Here, the surface area of the nucleus is presented at distance *r *= 1 µm. The insets of Fig. 3 and Figure S5 depict the average values of the geometric parameters as a function of the calcium concentration. Trend curves (red) were fitted to these data with standard statistically relevant distributions using a first to third smoothening spline function.

#### Image controls. Analysis of deconvolved fluorescent beads in a gel

We employed a control sample consisting of 1-μm-diameter fluorescent beads (Life Technologies, Waltham, MA, USA) embedded into a 4% polyacrylamide gel for testing our imaging system for optical aberrations and for quantification of the point-spread function in the deconvolution procedure of the 2D images. The bead sample was imaged in the same way (e.g., imaging depth) as for the keratinocytes and the full epidermis. The same image analysis strategy as for the granules was used to determine the localization and geometrical parameters of the beads.

### Colocalization analysis in NHEK cells

Colocalization image analysis in rat keratinocytes was performed using ImageJ software colocalization plug-in. Otherwise, colocalization analyses were performed on deconvolved and surface-rendered images employing the Pearson coefficient in Huygens. Values of close to 0 depict no and close to 1 full colocalization. Data retrieved were plotted in Prism as above; unpaired *t*-test was used for comparisons.

### Quantification of cornification by Laurdan imaging

Measurements of the plasma membrane stiffness were carried out on live NHEK monolayers, employing optical microscopy (assessment of spectral imaging on a Zeiss 780 inverted microscope, Zeiss, Jena, Germany), using 6-dodecanoyl-2-dimethylaminonaphthalene (LAURDAN; Sigma Aldrich, Gillingham, Dorset, UK). Fluorescence emission of Laurdan was excited at 374 nm and recorded over its whole spectrum from 405 to 600 nm. The intensity of emission wavelengths at 440 ± 10 nm and 490 ± 10 nm was used to obtain the generalized polarization (GP) value.

Values of GP vary from 1 to –1, where higher numbers reflect lower fluidity or higher stiffness, while lower numbers indicate an increase in fluidity. The images were then analyzed using a custom plug-in compatible with Fiji/ImageJ, and frequency histograms of the GP values were generated in Origin Pro (Northampton, MA, USA). A fit of a double Gaussian distribution to the distribution allowed determining average GP values, as well as standard deviations of both populations. The detailed technical description and its application have been gathered as a separate manuscript.

## Electronic supplementary material

Supplementary material(DOCX 19 kb)

Supplementary Figures(PDF 1440 kb)

Video File

Video File

Video File
