# Supplementary material for: Orchestrated control of filaggrin–actin scaffolds underpins cornification
Source: Cell Death Dis. 2018 Mar 15;9(4):412. doi: 10.1038/s41419-018-0407-2 (PMC5854575; doi:10.1038/s41419-018-0407-2)
Supplement: Supplementary file 1 — Supplementary material(DOCX 19 kb) [file 41419_2018_407_MOESM1_ESM.docx]

**Supplementary material**

**I. Supplementary Figure legends**

**Figure S1. Characterization of the antibodies recognizing profilaggrin/filaggrin epitopes expressed within KHGs**

(A) Western blot pattern of staining of epidermal sheet lysates blotted with 15C10, G-20 and H300 antibodies. PFLG – profilaggrin; FLG – filaggrin monomer ~37kDa. (B) Antibodies used in the study and their immunogens. (C, E, G) Two-color scanning confocal images of KHGs in fixed keratinocytes (NHEKs) subjected to low or high calcium level (0.06 and 1.5 mM as labeled) and immunolabeled for KHGs with two antibodies (as labeled). Scale bar 10µm; inset showing colocalisation maps. (D, F, H) Colocalisation analysis of the two-color images with values of the Pearson coefficient. Box and whisker representations and median values of the Pearson coefficient values of the two-color images of the respective two antibodies as labeled. A value of close to 1 indicates the expected congruent staining pattern. Analysis based on 3-5 cells scanned per condition; representative of 3 separate experiments.

**Figure S2. Filaggrin-containing keratohyalin granules undergo shape alterations and aggregation during formation of stratified epidermis – continued**

Geometric analysis of granule distribution within the epidermis (from scanning confocal images of Figure 1) based on the topological axial length parameter relative to normalized height. Black dots indicate granules smaller than 3 µm in length and red dots indicate granules larger than 3 µm in length (vertical dotted line indicates threshold); n=9170 granules in 120 cells in N=8 donors). The discrete length patterning in the lower epidermal layers arise from the restricted maximum 3D resolution imposed by sampling by the Software Object Analysis Tool, which is based on the PSF calibration obtained from fluorescent beads (see Methods).

**Figure S3. Bead controls.**

Control experiments using fluorescent beads. (Upper panels) 3D scanning confocal images (left: raw, right: deconvolved) of 1µm diameter fluorescent beads embedded in poly-acryl-amide gel. Microscope cover slide at bottom, x-y-z directions as indicated. (Lower panels) Values of axial length (upper graph; left: raw data; right: deconvolved data) and sphericity (lower graph; left: raw data; right: deconvolved data) of imaged beads as determined from the confocal images, scatter plotted against the distance (height) from the microscope cover slide, values from raw (left panels) and deconvolved (right panels) images (from n=5 images).

**Figure S4. Filaggrin expression in KHGs+ cells is promoted by differentiation.**

2D scanning confocal images of fixed normal human epidermal keratinocytes (NHEKs) immunostained for filaggrin (green, G-20 primary and Alexa Fluor 488 secondary antibody) and nucleus (blue, Hoechst) and grown in monolayers at different calcium levels. (A) Low ([Ca2+]=0.06mM, upper panel) and high ([Ca2+]=1.5mM, lower panel) calcium levels indicating low spontaneous (upper panel) and high differentiation-dependent (lower panel) expression of filaggrin (white arrows), respectively. Scale bar 20µm. (B) Further increasing levels of calcium (upper panels: [Ca2+]=1.5mM, middle panels: [Ca2+]=2.5mM, lower panels: [Ca2+]=5mM) indicating that filaggrin-containing granules undergo shape changes during calcium-induced keratinocyte differentiation. Images were not deconvolved to better visualize cytoplasmic filaggrin+ material; images 600µm above and below equatorial planes of the granules show material release (compare Figure 2J). Different panels at [Ca2+]=2.5mM (middle) depict 2D images taken at different axial (z) planes (as labeled). Scale bar 5µm. (C) 3D modelling of keratohyalin granules labelled with N-terminal specific anti-filaggrin antibody (15C10; Alexa 488). Data analysis based on 500-1000 granules per cell. NHEKp4 – normal human epidermal keratinocytes (passage 4); red-purple: pseudo-colour for Hoechst.

**Figure S5. Filaggrin-containing granules undergo shift in spatial distribution and locate in the proximity and within the limits of cell nucleus (further statistical data)**

(A)–(B) Characterisation of KHG population from confocal scanning microscopy data of fixed NHEK cells at different calcium switches i.e. different differentiation stages of figure 2. (A) Granule axial length, (B) lateral aspect ratio (length vs maximum width, a value close to 1 indicates spherical granules), and (C) axial aspect ratio (length vs minimum width, difference vs lateral aspect ratio indicates more complex granule shape) as a function of the distance to the nuclear membrane. Colour coding: cold colours/low calcium = low differentiation state; warm colours/high calcium = high differentiation state (see also colour bar). Reference point of granules locations (nuclear membrane) denoted with dashed line at l=1). The distance |r| is the absolute value of the differences of granule spatial coordinates while granules located within the nucleus had at least one negative spatial coordinate (x, y, or z). Insets: dependence of the average value of the respective parameter on calcium concentration [Ca2+]; > 50 cells analysed; error bars = SD.; p<0.01, n=11152 in ~50 cells analysed; data representative of 3 separate experiments. The discrete length patterning in panel a arise from the restricted maximum 3D resolution imposed by sampling by the Software Object Analysis Tool, which is based on the PSF calibration obtained from fluorescent beads (see Methods).

**Figure S6. “Granule maturation” in live keratinocytes undergoing differentiation.**

(A) Phase contrast and 2D scanning confocal images (left, middle: overlay; right: confocal) of live keratinocytes intracellularly labeled for filaggrin (lower panels, G-20 antibody, Alexa Fluor 488 secondary antibody) or isotype control antibody (upper panels, IsoC isotype control and secondary Alexa Fluor 488 antibody) before (left panels) and 24h after (middle and right panels) “calcium switch” ([Ca2+]=1.5mM). Arrows represent cells containing granules positive for filaggrin staining. (B) Scanning confocal images of live keratinocytes intracellularly labeled for filaggrin (green, G-20 as above) and nuclei (red, Hoechst) over a time course of 48h as labeled. Scale bar 20µm. Data representative of 5 separate experiments. (C-E) Mean values of the granules’ geometric parameters over time as determined from the live-keratinocyte confocal recordings (C) Granule volume, (D) surface area and (E) axial length. Vertical dotted line represents calcium switch at 24h.

**Figure S7. Collapse of actin cytoskeleton during keratinocyte differentiation and cornification.**

**(**A) 2D scanning confocal images of fixed NHEKs immunostained for F-actin (green; phalloidin-488) 24h after “calcium switch” with different calcium levels [Ca^2+^] = 0.06 – 0.5 mM as labelled. Insets: Zoom-ins of areas marked by the white box in the respective overview images, depicting changes in integrity of actin cortex filaments (white arrows) and F-actin localization within and in the proximity of the nucleus (red arrows). Scale bar 10µm. Representative of 2 separate experiments. (B) 2D confocal scanning image of fixed NHEK at [Ca^2+^] = 2.5 mM immunostained for F-actin (green, phalloidin staining) and the nucleus (blue, Hoechst), depicting F-actin rings in the direct proximity and within the nucleus. Scale bar 10µm. Representative of 2 separate experiments.

**Figure S8. Actin colocalisation with filaggrin.**

(A) 2D scanning confocal images of fixed keratinocytes (NHEKs) immunostained for filaggrin (green, G-20 and secondary Alexa Fluor 488), nucleus (blue, Hoechst) and α-actin (red, upper panel, 1A4, Alexa Fluor 568 secondary antibody), β-actin (red, middle panel, 4C2, Alexa Fluor 568 secondary antibody) and γ-actin (red, lower panel, and 2A3, Alexa Fluor 568 secondary antibody) and subject to low ([Ca2+]=0.06mM, left panels) and high calcium levels ([Ca2+]=0.15mM, right panels). Scale bar 10µm; inset showing colocalisation maps. (B) Box and whisker plots and median values of the Pearsons coefficient of colocalisation of filaggrin and α-actin (upper), β-actin (middle) and γ-actin (lower panel) from the two-color images for the two calcium conditions as labelled. A value of close to 1 indicates an overlap of both staining patterns. Analysis based on 3-5 cells scanned per condition; representative of 3 separate experiments; unpaired t test used for comparisons. Scale bar 10µm.

**Figure S9. Filaggrin vs actin colocalisation and actin cytoskeleton dependence on keratinocyte differentiation (pooled data represented in Figure S8).**

Change in colocalisation between filaggrin and actin isoforms staining (α-, β- and γ-actin as labeled) during keratinocyte differentiation as determined from the confocal images on fixed NHEKs. Calculation of fold change in Persons coefficient between low and high calcium levels is represented; mean with SEM shown; unpaired t test used for comparisons pooled data as represented in Figure S8.

**Figure S10. Actins form granule-associated scaffold structures – raw data**

Two-color raw 3D STED images of Figure 5: fixed NHEKs 24h after “calcium switch” ([Ca2+]=1.5mM) immunostained for filaggrin (green, G-20 primary antibody, Alexa Fluor 488 secondary antibody) and α-actin (red, left panel; 1A4 antibody, Alexa 568 secondary) and β-actin (red, right panel, 4C2 antibody, Alexa 568 secondary): 2D overviews (main panels; scale bar 10µm) and 3D zoom-ins on representative volumes (insets). Data representative of 3 separate experiments.

**Figure S11. Lack of colocalisation between filaggrin and γ-actin actin revealed by STED.**

Representative 3D-STED image of fixed NHEKs grown at high calcium level ([Ca2+]=1.5mM) and immunostained for filaggrin (green) and γ-actin (2A3, red); large overview (B, scale bar 10µm) and zoom-in (C, scale bar 2µm) indicating very abundant cytosolic presence but little colocalisation with filaggrin granules of γ-actin; representative of 3 separate experiments.

**Figure S12. LatB and CytD disrupts actin cytoskeleton over short timescales.**

(A, B) 2D scanning confocal images of fixed NHEKs immunostained for F-actin (green, phalloidin-488) and nucleus (blue, Hoechst) following no (left, DMSO) and actin disruption treatment by latrunculin B (LatB, 5-min treatment middle left panel, 60-min middle right, as labelled) and cytochalasin D (Cyt D, 5-min treatment second panel from right, 60-min right panel, as labelled) at low (A, [Ca2+]=0.06mM) and high (B, [Ca2+]=0.06mM) calcium levels; data representative of 2 experiments. Scale bar 10µm.

**II. Supplementary Movies**

**Movie 1: Filaggrin-containing granule distribution and morphologies in differentiated keratinocyte.**

3D reconstruction of high resolution confocal scanning of normal human epidermal keratinocyte (NHEK) labeled for filaggrin. The cells were subjected to calcium switch at [Ca2+]=1.5mM for 24h, fixed and immuno-labeled (G-20 antibody and Alexa Fluor 488 secondary antibody; Hoechst staining for nucleus). White arrows: ring-like granule presentation in nuclear proximity; yellow arrows: tubular granule presentation; red arrow: release of filaggrin+ material within or near keratinocyte nucleus; grey arrow: release of filaggrin+ material in the proximity of the nucleus. Scale bar 20µm; data represented of 3 separate experiments.

**Movie 2: Filaggrin-containing granule distribution and morphologies undergo morphological changes and aggregate in live keratinocytes subjected to “calcium switch”.**

3D reconstruction of confocal scanning of normal human epidermal keratinocyte (NHEK) during 24h post 0.06mM to 1.5mM calcium switch (“first switch”). Normal human keratinocytes internally immunolabeled live for filaggrin (G-20 antibody and Alexa Fluor 488 secondary antibody) at low calcium level; Hoechst staining for nucleus. White arrows: granule aggregates; red arrow: granule aggregate releasing filaggrin^+^ material; Scale bar 20µm. Data representative of 5 separate experiments.

**Movie 3: Filaggrin-containing granule distribution and morphologies undergo morphological changes and aggregate in live keratinocytes subjected to “calcium switch”.**

3D reconstruction of high resolution confocal scanning of normal human epidermal keratinocyte (NHEK) during 24h period post 1.5mM to 5mM calcium switch (“second switch”; “first switch represented in Movie 2). Normal human keratinocytes grown internally immunolabeled live for filaggrin (G-20 antibody and Alexa Fluor 488 secondary antibody; Hoechst staining for nucleus. White arrows: granule aggregates; red arrow: intrusion of granule aggregate into the nucleus and subsequent nucleus degradation; Scale bar 20µm. Data representative of 5 separate experiments.

**III. Supplementary material – supplementary Text**

**1. Live imaging of keratinocyte differentiation after step-wise calcium switch**

In order to confirm the results obtained in previous experiments by observing the “granule maturation” as a dynamic process over time we developed a system to detect those changes in live keratinocytes at a single cell level. Adapted cationic lipid-aided intracellular staining protocol described by Weill et al.^68^ was used for studying differentiation on live keratinocytes (Figure S6). We labelled live primary keratinocytes with filaggrin antibody/secondary antibody pairs at low calcium concentration ([Ca2+]=0.06mM). Staining of those undifferentiated cells revealed very little specific staining (“dot positivity” characteristic of profilaggrin expression in the form of granules), and expected relatively high background level, as a result of antibody dispersion in the cytoplasm (Figure S6A). We then carried out live-cell imaging after a 48h-long “calcium switch” experiments, in which we exposed internally labelled keratinocytes to [Ca2+]=1.5mM for the initial period of 24h and thereafter to another calcium switch at up to [Ca2+]=5mM calcium concentrations.

We recorded 3D scanning confocal images on these live keratinocytes every hour throughout a 48h-long experiment and determined changes in granule morphology and localization from the 3D-rendered images. We observed an increase in staining of granules visualized as “dot positivity” (a characteristic staining pattern representing filaggrin-containing granules), coinciding with a reduction in background staining levels. This was in line with the staining of nascent profilaggrin-containing granules, i.e. increase in dot number was indicative of formation of new KHGs (Figure S6A). The observed morphological changes were continuously more pronounced with increasing levels of calcium concentration, specifically with respect to granule length, surface area, number, and volume (Figure S6B-C, Movies 2-3).

**2. Actin cytoskeleton during late keratinocyte differentiation**

To investigate the potential role of the actin cytoskeleton during KHG maturation, we employed the *in vitro* “calcium switch” model to investigate this further. Using confocal microscopy on fixed NHEK cells, we found that filamentous actin (F-actin, labelled with phalloidin) undergoes dramatic changes in keratinocytes within the range of physiologically-relevant calcium concentrations (Figure 4). Specifically, cellular actin cortex lost structural integrity and fully collapsed at high calcium concentrations (from [Ca2+]=1.5mM). Interestingly, at this and higher calcium levels, F-actin also accumulated within the nucleus, displaying punctate and diffuse actin foci-like appearance at [Ca2+]=1.5-2.5mM and 5mM, respectively. Remarkably, at [Ca2+]=2.5mM we were able to observe F-actin rich rings in the close proximity of the nucleus in a small (5%) fraction of cells (Figure 4B); their appearance mirrored the ring-like granules observed before at this differentiation stage (compare Figure 2I-J).

Actins exert multiple cellular functions; it is recognized that α- and γ2-actin isoforms are predominantly present in sarcomeres of muscle cells while both β- and cytoplasmic (non-muscle) γ1-actin are ubiquitously expressed in other cell types^40^. To elucidate whether there is a direct molecular interaction between actin and granules, we examined all three actin homologues and performed co-localization analysis for filaggrin and actins in fixed NHEKs. Using multi-color fluorescent confocal microscopy, we found high constitutive co-localization of α-actin with filaggrin-labelled KHGs and significant increase in co-localization prompted by calcium for the β- and non-muscle γ-actin isoforms ([Ca2+]=1.5mM; Supplementary Figure S8 and Figure S9).
