# Supplementary material for: Orchestrated control of filaggrin–actin scaffolds underpins cornification
Source: Cell Death Dis. 2018 Mar 15;9(4):412. doi: 10.1038/s41419-018-0407-2 (PMC5854575; doi:10.1038/s41419-018-0407-2)
Supplement: Supplementary file 2 — Supplementary Figures(PDF 1440 kb) [file 41419_2018_407_MOESM2_ESM.pdf]

II. Supplementary Figures

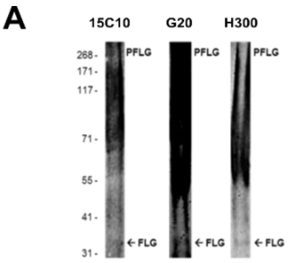

B

FILAGGRIN ANTIBODY	IMMUNOGEN	SPECIES
H-300	1-300 (h)	Rb
G-20	internal (h) 330-380	Gt
15C10	N-terminus (h)	Ms

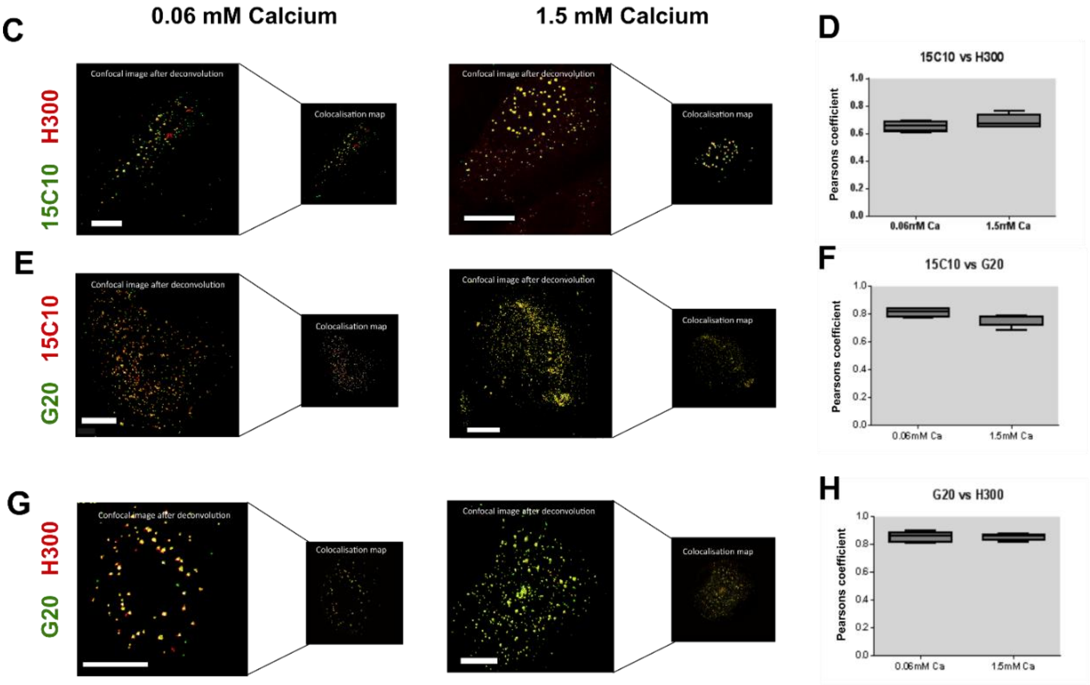

Figure S1.

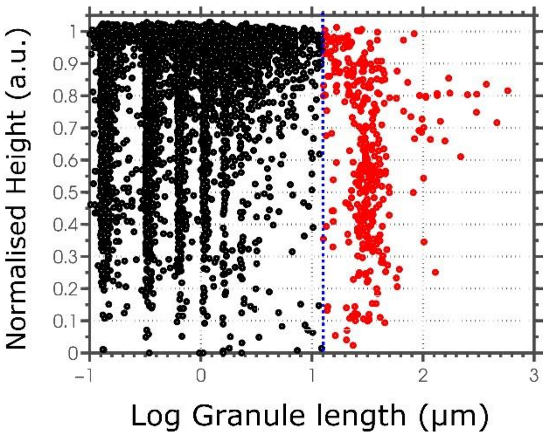

Figure S2.

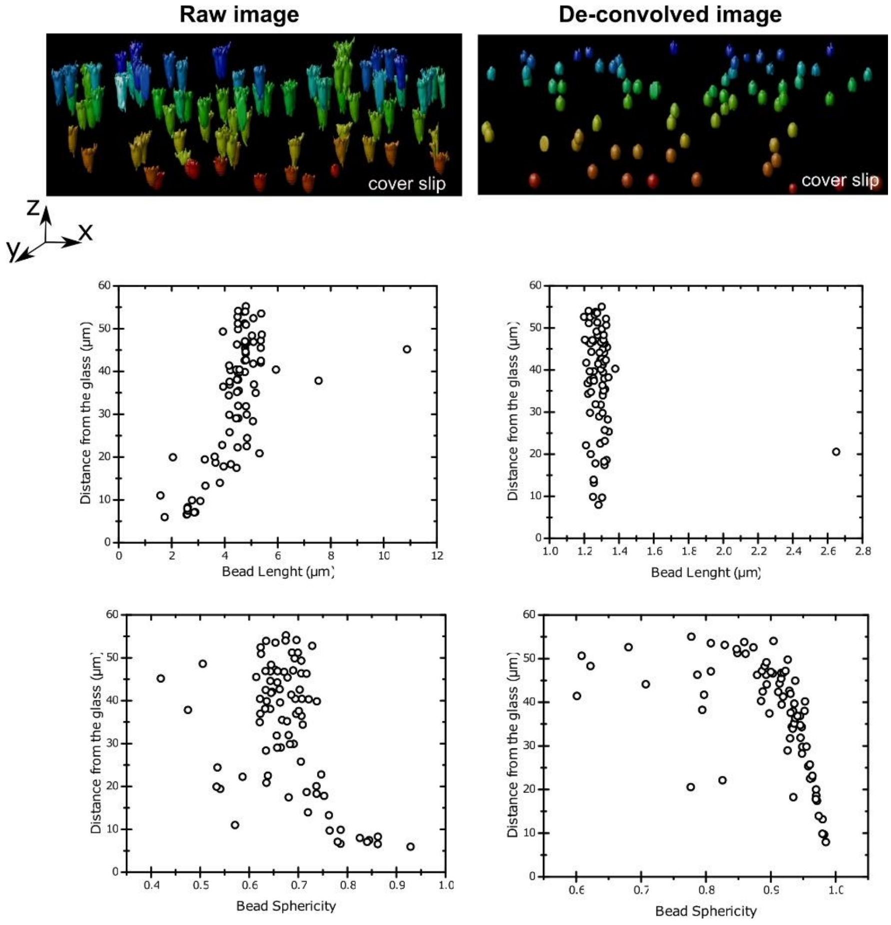

Figure S3.

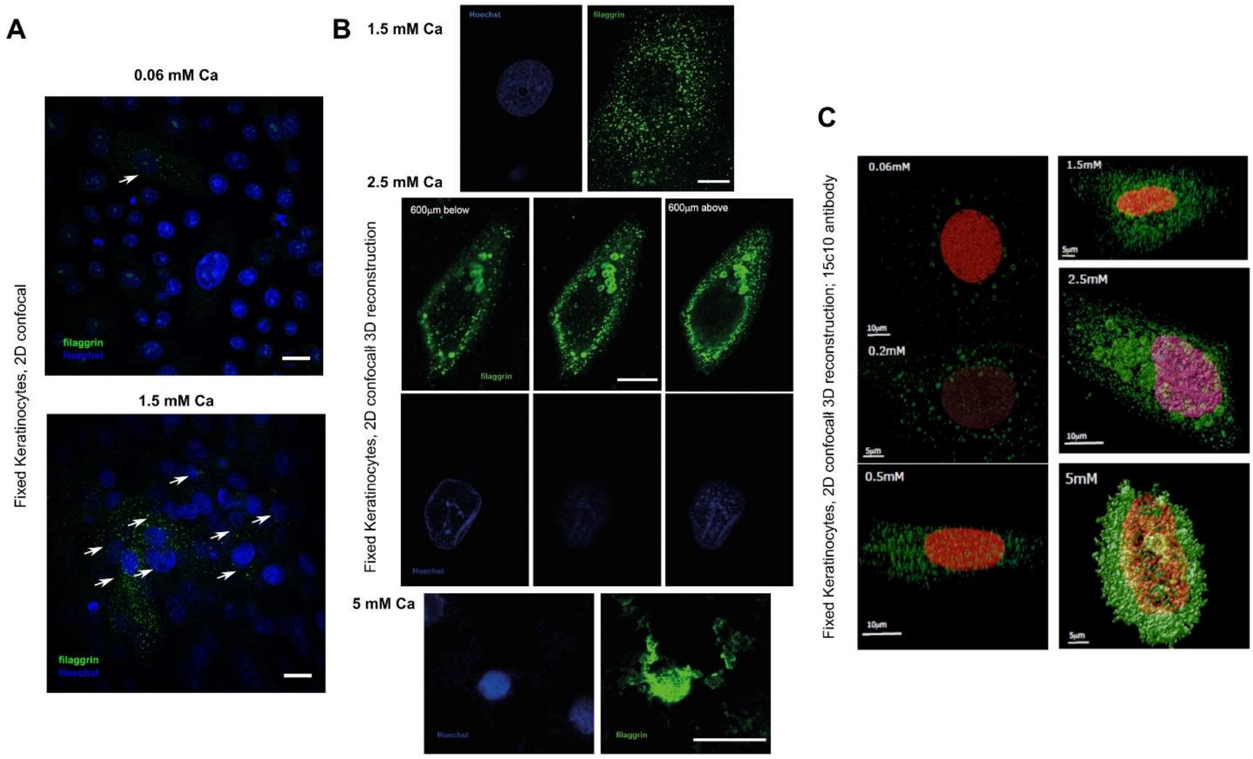
A
0.06 mM Ca
filaggrin
Hoechst
1.5 mM Ca
filaggrin
Hoechst
Fixed Keratinocytes, 2D confocal
B
1.5 mM Ca
Hoechst
filaggrin
2.5 mM Ca
600μm below
600μm above
filaggrin
Hoechst
Fixed Keratinocytes, 2D confocal 3D reconstruction
5 mM Ca
Hoechst
filaggrin
C
0.06mM
10μm
0.2mM
5μm
0.5mM
10μm
1.5mM
5μm
2.5mM
10μm
5mM
5μm
Fixed Keratinocytes, 2D confocal 3D reconstruction; 15c10 antibody

Figure S4.

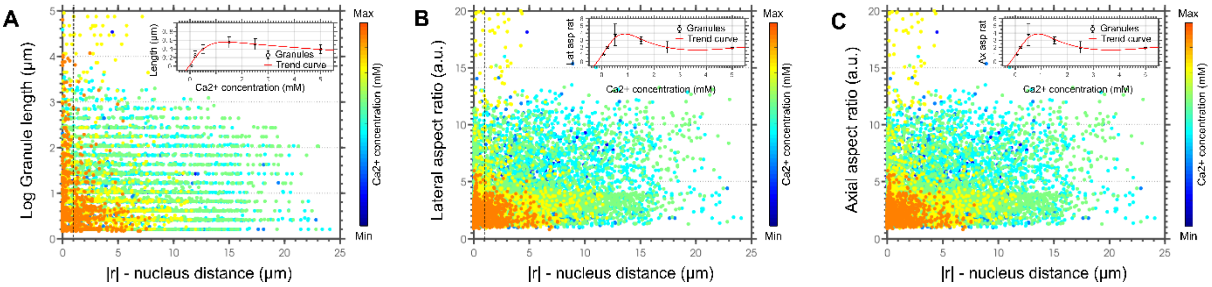

Figure S5.

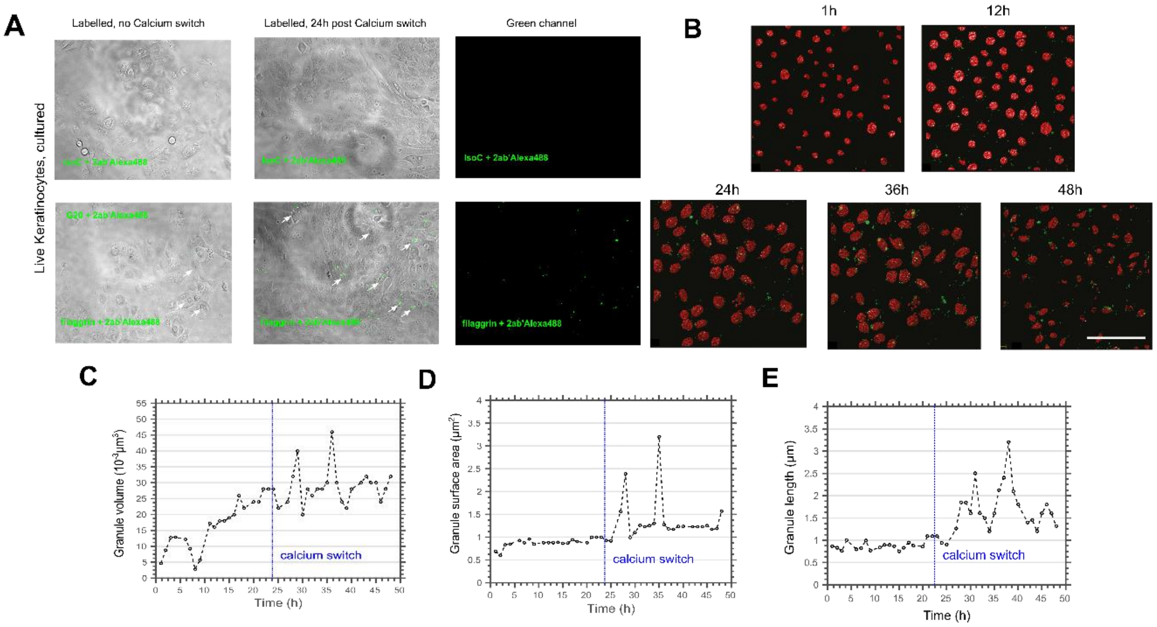

Figure S6.

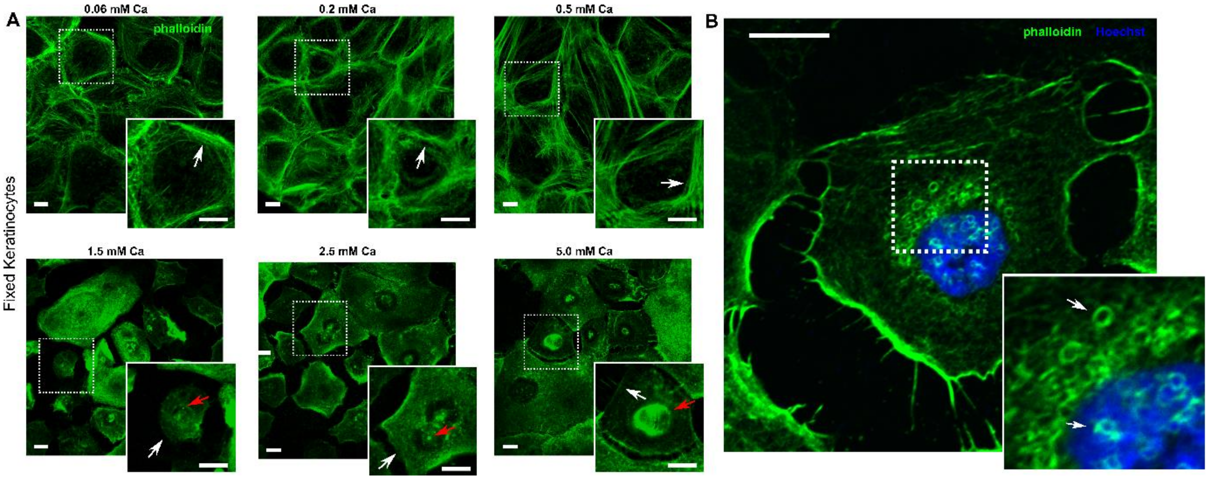

Figure S7.

Figure S8.

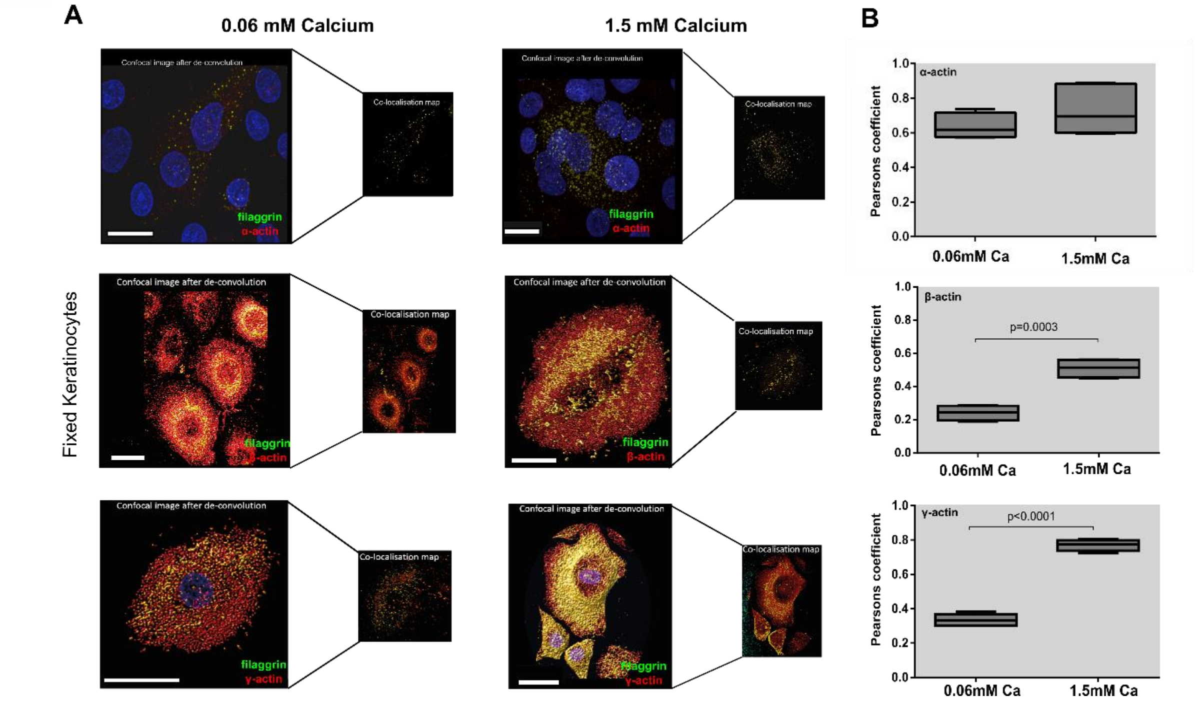

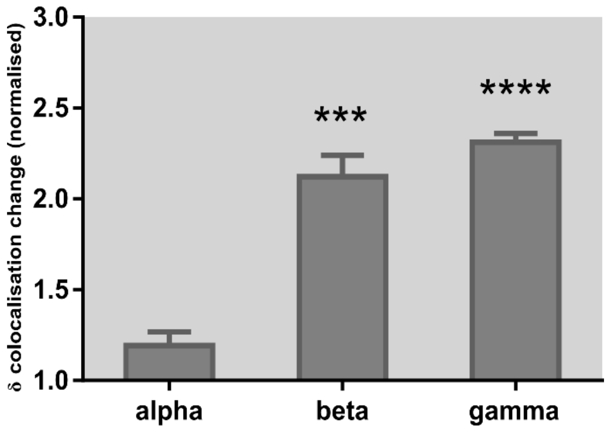

Figure S9.

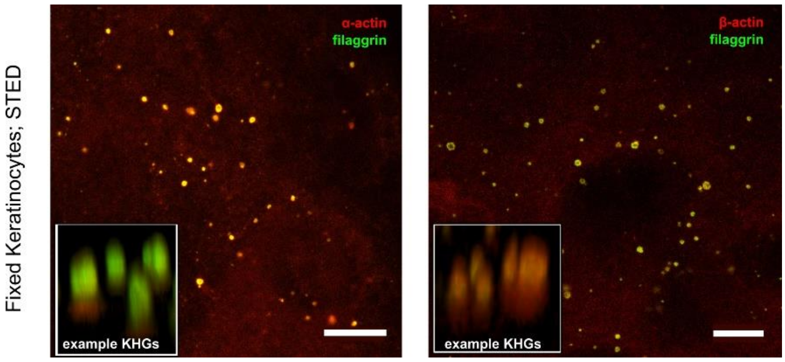

Figure S10.

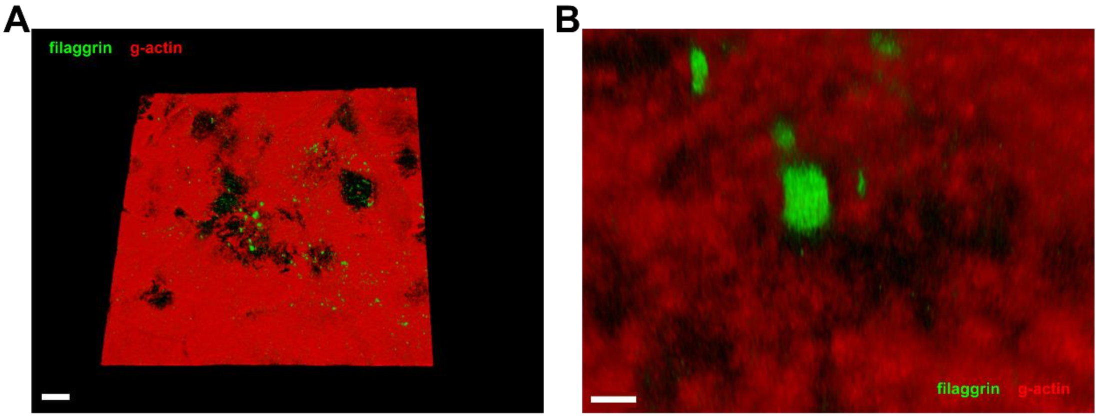

Figure S11.

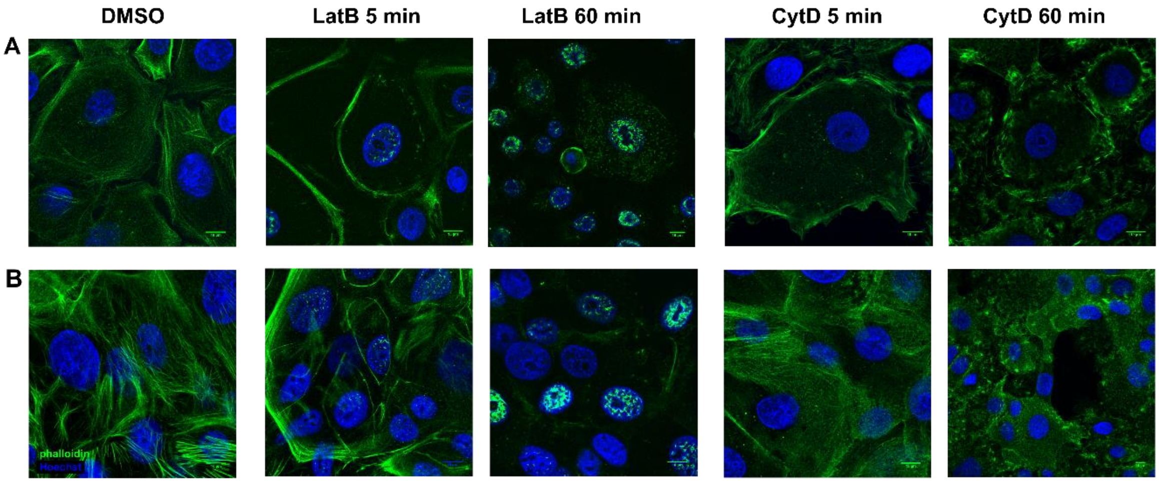

Figure S12.
